# Telomere-to-telomere genome assembly of asparaginase-producing *Trichoderma simmonsii*

**DOI:** 10.1186/s12864-021-08162-4

**Published:** 2021-11-17

**Authors:** Dawoon Chung, Yong Min Kwon, Youngik Yang

**Affiliations:** National Marine Biodiversity Institute of Korea, Chungchungnam-do, South Korea

**Keywords:** *Trichoderma simmonsii*, Telomere-to-telomere assembly, Comparative genome analysis, Asparaginase

## Abstract

**Background:**

*Trichoderma* is a genus of fungi in the family Hypocreaceae and includes species known to produce enzymes with commercial use. They are largely found in soil and terrestrial plants. Recently, *Trichoderma simmonsii* isolated from decaying bark and decorticated wood was newly identified in the Harzianum clade of *Trichoderma*. Due to a wide range of applications in agriculture and other industries, genomes of at least 12 *Trichoderma* spp. have been studied. Moreover, antifungal and enzymatic activities have been extensively characterized in *Trichoderma* spp. However, the genomic information and bioactivities of *T. simmonsii* from a particular marine-derived isolate remain largely unknown. While we screened for asparaginase-producing fungi, we observed that *T. simmonsii* GH-Sj1 strain isolated from edible kelp produced asparaginase. In this study, we report a draft genome of *T. simmonsii* GH-Sj1 using Illumina and Oxford Nanopore technologies. Furthermore, to facilitate biotechnological applications of this species, RNA-sequencing was performed to elucidate the transcriptional profile of *T. simmonsii* GH-Sj1 in response to asparaginase-rich conditions.

**Results:**

We generated ~ 14 Gb of sequencing data assembled in a ~ 40 Mb genome. The *T. simmonsii* GH-Sj1 genome consisted of seven telomere-to-telomere scaffolds with no sequencing gaps, where the N50 length was 6.4 Mb. The total number of protein-coding genes was 13,120, constituting ~ 99% of the genome. The genome harbored 176 tRNAs, which encode a full set of 20 amino acids. In addition, it had an rRNA repeat region consisting of seven repeats of the 18S-ITS1–5.8S-ITS2–26S cluster. The *T. simmonsii* genome also harbored 7 putative asparaginase-encoding genes with potential medical applications. Using RNA-sequencing analysis, we found that 3 genes among the 7 putative genes were significantly upregulated under asparaginase-rich conditions.

**Conclusions:**

The genome and transcriptome of *T. simmonsii* GH-Sj1 established in the current work represent valuable resources for future comparative studies on fungal genomes and asparaginase production.

**Supplementary Information:**

The online version contains supplementary material available at 10.1186/s12864-021-08162-4.

## Background

Fungal species belonging to the genus *Trichoderma* produce a variety of valuable factors with different function, including enzymes [1]. For example, *T. reesei* is a representative cellulolytic microorganism used for the degradation of lignocellulosic plant materials. Cellulases from *T. reesei* and *T. viride* as well as chitinase, xylanase, and lysine oxidase from *T. viride* are already commercially available [2, 3].

In addition to enzymatic activities, various *Trichoderma* fungi have been extensively studied for their mycoparasitic properties. In particular, *T. harzianum* is used as a commercial biocontrol agent against plant diseases [4]. In fungal phylogeny, the Harzianum clade consists of at least 18 *Trichoderma* species, including *T. harzianum*, *T. guizhouense*, *T. inhamatum*, *T. lentiforme*, *T. lixii*, *T. afarasin*, *T. afroharzianum*, *T. atrobrunneum*, *T. camerunense*, *T. endophyticum*, *T. neotropicale*, *T. pyramidale*, *T. rifaii*, *T. simmonsii* [5], *T. lentinulae*, *T. vermifimicola*, *T. xixiacum*, and *T. zelobreve* [6]. Fungi in this clade are ubiquitous and often isolated from the soil as well as plant debris and occasionally from marine resources such as sediments and sea sponges [7, 8].

*T. simmonsii* is a newly described fungal species belonging to the Harzianum clade, mostly isolated from decaying bark and decorticated wood [5]. Since its first identification in the United States in 2015 (MycoBank MB809947), *T. simmonsii* strains have been reported in several countries in Europe and, more recently, in China and South Korea [9, 10]. This fungus was also identified in formulated biocontrol agents [5]. Furthermore, *T. simmonsii* strain UTFC 10063 efficiently accumulates cadmium in its biomass, exhibiting potential as a bio-removal agent in cadmium-polluted solutions [11]. However, when compared to other *Trichoderma* species, the molecular characteristics and bioactivities of *T. simmonsii* are poorly understood.

Fungal genome analyses have highlighted the genetic diversity within the fungal kingdom in addition to differences in fungal morphology, physiology, and ecology [12]. Due to advances in high-throughput sequencing technologies, the body of available fungal genome data is rapidly increasing. Recently, genomes of the most common 12 *Trichoderma* spp. including *T. reesei*, *T. parareesei*, *T. longibrachiatum*, *T. citrinovirde*, *T. harzianum*, *T. afroharzianum*, *T. guizhouense*, *T. virens*, *T. asperellum*, *T. hamatum*, *T. atroviride*, and *T. gamsii*, were compared in order to understand the evolution, core genome, and gene inventory of *Trichoderma* [13].

Our laboratory has screened different marine-derived fungi with enzymatic activities. L-asparaginase (L-asparagine amidohydrolase, EC 3.5.1.1) is an enzyme that hydrolyzes L-asparagine to aspartic acid and is utilized for the treatment of acute lymphoblastic leukemia [14] as well as for the reduction of carcinogenic compound acrylamide in food [15]. Currently, asparaginases from *Escherichia coli* and *Erwinia chrysanthemi* are utilized as therapeutic agents [16]. However, the discovery of novel asparaginases is necessary as bacterial asparaginase occasionally causes adverse effects, including allergic responses. In this study, we report the genomic analysis of marine-derived *T. simmonsii* isolate GH-Sj1, one of the fungal strains we screened for asparaginase activity. In addition, we performed transcriptomic analysis of GH-Sj1 under asparaginase-rich conditions. Although *Trichoderma* species are well-known valuable resources for industrial enzymes, their asparaginase production remains unexplored. Through this paper, we provide insights into the *T. simmonsii* genome as well as its expression profile under asparaginase-rich conditions.

## Results

### Identification of marine-derived *T. simmonsii* GH-Sj1

**Fig. 1:**
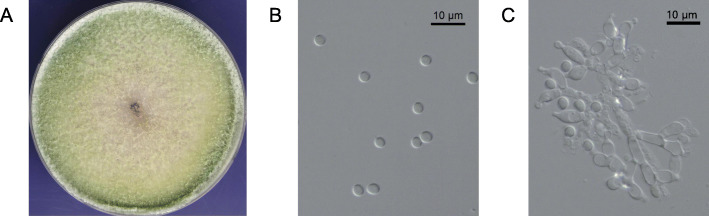
Characterization of *Trichoderma simmonsii* GH-Sj1 morphology. (**A**) Colony of GH-Sj1 cultured on PDA at 25 °C for 7 days. (**B**) Microscopic images of conidia. GH-Sj1 produces subglobose to ovoid conidia. (**C**) A conidiophore image of GH-Sj1

A marine-derived strain, designated GH-Sj1, was isolated from a sea algae *Saccharina japonica* collected in Sacheon, Republic of Korea. This strain produced abundant aerial mycelia and whitish and green granular colonies on PDA at 25 °C for 7 days (Fig. 1A). It produced subglobose to ovoid conidia in a green disk around the inoculum with sizes in the range of 2.5–3.0 μm in width × 2.8–3.5 μm in length (*N* = 10) (Fig. 1B). Conidiophores developed to form branches having a terminal whorl of multiple phialides (Fig. 1C). These morphological features of GH-Sj1 were similar to those of *Trichoderma spp.* previously reported [5].

Molecular identification was performed using a translation elongation factor 1α gene (*tef1α*) sequence that is a widely used genetic marker for *Trichoderma* identification [17, 18]. From the BLASTN search, *tef1α* of GH-Sj1 showed a high degree of sequence identity to that of the *T. simmonsii* type specimen G.J.S. 91–138 (98.8%, GenBank AF443935). In addition, GH-Sj1 *tef1α* sequence was also similar to that of *T. lentinulae* CGMCC 3.19847 (94.8%, GenBank MN605878), *T. camerunense* GJS 99–230 (94.0%, GenBank AF348107), and *T. harzianum* CBS 226.95 (92.8%, AF348101). Consequently, based on the morphological and molecular features, GH-Sj1 was identified as *T. simmonsii*.

### DNA sequencing of *T. simmonsii* isolate GH-Sj1

To reconstruct the high quality genome of *T. simmonsii* GH-Sj1, we generated sufficient sequencing depth of Illumina short reads for high base accuracy [19] and Nanopore long reads to overcome fragmented assembly [20, 21]. As a result, we obtained, in total, 7,521,311,812 bp from 24,905,006 PE reads using Illumina Technologies’ short read sequencing platform, which resulted in a genome coverage of ~187x. After trimming low-quality bases, 6,985,160,902 bp from 23,255,700 PE reads were retained, corresponding to ~174x genome coverage. Utilizing the Oxford Nanopore Technologies’ long read sequencing platform, 795,128 long reads (7,510,994,507 bp) were generated, with a genome coverage of ~187x. Finally, 795,176 reads (7,480,287,989 bp) remained after adapter trimming, covering ~186x of the genome length.

### Genome assembly of *T. simmonsii* isolate GH-Sj1

Since short length reads often leads to fragmented de novo assembly [21], we only used 239,681 Nanopore reads (5,783,154,314 bp) with at least 10 Kb for the genome assembly which constituted a genome coverage of ~144x. We then compared the performances of multiple de novo assemblers in order to find the best draft assembly, which included Canu [22], Flye [23], Miniasm [24], Shasta [25], and Wtdbg2 (v2.3) [26]. These draft assemblies were polished using Nanopore long reads and Illumina short reads as described in the Methods section.

**Fig. 2:**
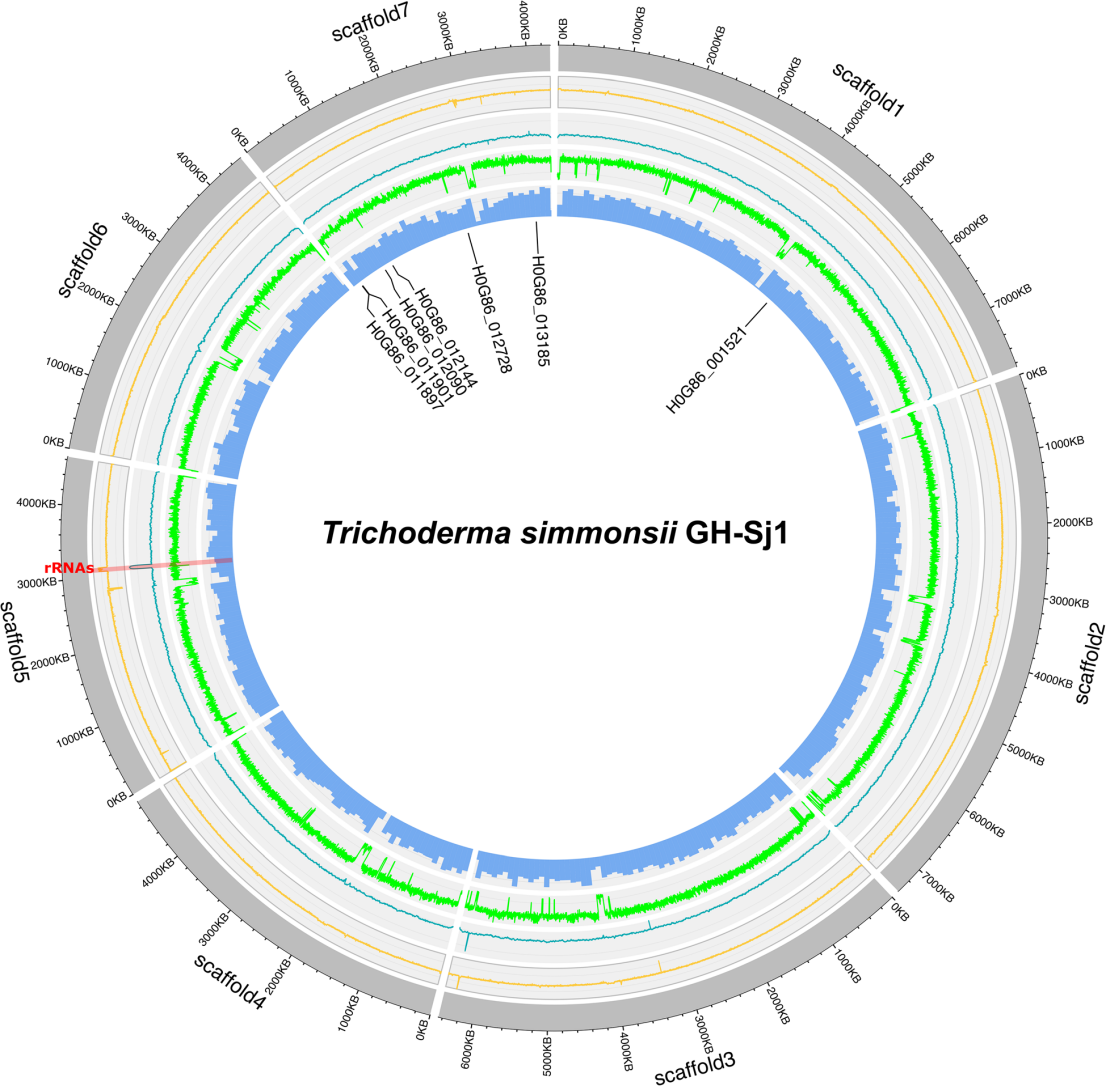
Circular representation of the *Trichoderma simmonsii* GH-Sj1 genome. From outer ring to inner ring: first ring, scaffolds; second ring, log2 of Illumina WGS read coverages in 1 Kb windows; third ring, log2 of Nanopore WGS read coverages in 1 Kb windows; fourth ring, GC contents in 1 Kb windows; fifth ring, gene counts in 100 Kb windows. The tracks and labels of genes above the title: the locus of asparaginases

Supplementary Table 1 shows the results of the five draft assemblies. The assembly lengths, GC contents and BUSCO scores were comparable among the assemblers, where the values were approximately 40 Mb, 48, and 99%, respectively. However, Miniasm output was best by several criteria. It generated the smallest number of contigs of 9, of which 7 contigs were longer than 100Kb. L50 was the best with Flye and Wtdbg2 at 3. Moreover, five contigs were assembled telomere-to-telomere. We therefore chose Miniasm contigs as the primary assembly and refined the results as follows. Two overlapping contigs were merged as one scaffold. Conversely, a contig was splitted which assembled to two contigs in other assemblers. In addition, we dropped a very short contig and a mitochondrial sequence. For more details, refer to the Methods section. As a result, the final genome assembly consisted of seven genomic scaffolds (40,078,385 bp) with an N50 length of 6.4 Mb. The *T. simmonsii* genome contained no gaps, and the GC content was 48.13% as summarized in Table 1. The average base coverage of genomes for Illumina WGS reads and Nanopore WGS reads were ~ 168x and ~ 186x, respectively. There were a couple of regions where read coverages were exceptionally high. One of these was the rRNA repeat region in scaffold 5, where the maximum base coverage of Illumina and Nanopore reads was 4704x and 9668x, respectively. All seven scaffolds were in chromosome scale, wherein each scaffold is assembled telomere-to-telomere. Table 1 shows the occurrences of telomere repeats for each scaffold. The minimum telomere repeats were 12, and the maximum were 17. For the full alignments of telomere regions, refer to Supplementary Table 2. As pointed out in [27], telomere regions are highly AT-rich, illustrated in Fig. 2 as the fourth ring from outside to inside. Centromere regions are also very high in AT and scarce in genes. In terms of centromere positions, scaffold 1, 2, and 6 appeared to be metacentric where p and q arms are of compatible length, while the others were submetacentric, with the p arm being shorter than the q arm. For assembly completeness, our genome assemblies recovered ~ 99% of the BUSCO [28] with regard to fungi_odb10 gene groups.

**Table 1:** Telomere repeat occurrences of *Trichoderma simmonsii* GH-Sj1

Terminus	Scaffold1	Scaffold 2	Scaffold 3	Scaffold 4	Scaffold 5	Scaffold 6	Scaffold 7
3′ ➔ 5′ (CCCTAA)*n*	13	12	14	12	12	17	13
5′ ➔ 3′ (TTAGGG)*n*	13	14	15	15	12	15	16

### Genome annotation of *T. simmonsii* isolate GH-Sj1

After achieving the high quality assembly of *T. simmonsii* GH-Sj1, we annotated the genome using funannotate pipeline which specialized for annotating fungal genomes [29]. As summarized in Table 2, 13,120 protein-coding genes (13,875 proteins) were predicted based on ab initio prediction and RNA-seq data. The number of predicted genes of *T. simmonsii* were similar as for other *Trichoderma* species (Table 3). The average gene length was 1452 bp, average exon length was 645 bp, and the average number of exons per gene was 2.96 (Table 2). The *T. simmonsii* genome consisted of 176 tRNA genes where the full set of 20 amino acids were found along with three defined as tRNA-iMet (Supplementary Table 3). Scaffold 5 contained an rRNA repeat region, highlighted in Fig. 2, where 21 rRNAs were predicted. The region harbored seven repeats of rRNA clusters, with each repeat consisting of an 18S-ITS1–5.8S-ITS2–26S rRNA gene cluster. Clusters of Orthologous Groups of proteins (COG) [30] functional category distribution is shown in Table 4 and Supplementary Fig. 1. Disregarding (S) Function unknown, the top 5 functional categories were (Q) Secondary metabolites biosynthesis, transport and catabolism, (G) Carbohydrate transport and metabolism, (O) Posttranslational modification, protein turnover, chaperones, (E) Amino acid transport and metabolism, and (U) Intracellular trafficking, secretion, and vesicular transport. *T. simmonsii* was most annotated in all the COG categories except for (V) Defense mechanisms, where it was second only to *T. harzianum*. Carbohydrate-Active Enzymes (CAZyme) [31] classification results are presented to Supplementary Fig. 2 and 3. The occurrences of CAZyme categories Auxiliary Activities (AA), Carbohydrate-Binding Modules (CBM), Carbohydrate Esterases (CE), Glycoside Hydrolases (GH), Glycosyl Transferases (GT), and Polysaccharide Lyases (PL) were 126, 12, 56, 423, 78, and 13, respectively. The top 10 most occurring families were CE10 (31), GH18 (24), AA7 (23), AA3 (20), GH3 (19), AA3_3 (19), AA3_2 (19), GH16 (16), GH2 (13), and GH55 (12), where the value in parenthesis represents the occurrence of each family. The antiSMASH results [32] are summarized in Supplementary Table 4, where the two most abundant cluster types were Type 1 Polyketide synthase (T1PKS) and Non-ribosomal peptide synthetase cluster (NRPS).

**Table 2:** Genome statistics of *Trichoderma simmonsii* GH-Sj1

Number of genomic scaffolds	7
Total length (bp)	40,078,385
N50 (bp)	6,451,197
Number of N’s (bp)	0
GC content (%)	48.13
BUSCO (genome)	98.7%
tRNA genes	176
rRNA genes	21
Number of protein coding genes	13,120
Number of proteins	13,875
Average gene length (bp)	1452.7
Average exon length (bp)	645
Average number of exons per gene	2.96

**Table 3:** *Trichoderma* genomes statistics

Clade	Species	Strain	NCBI accession	Genome size (Mb)	Sca-ffold	GC (%)	Genes	Proteins
*Harzianum/Virens*	*T. simmonsii*	GH-Sj1	N/A	40.07	7	48.13	13,296	13,875
	*T. guizhouense*	NJAU 4742	GCA_002022785.1	38.32	63	49.56	11,255	11,255
	*T. harzianum*	CBS 226.95	GCF_003025095.1	40.98	532	47.58	14,269	14,065
	*T. lentiforme*	CFAM-422	GCA_011066345.1	38.31	47	49.65	12,978	12,978
	*T. virens*	Gv29–8	GCF_000170995.1	39.02	93	49.25	12,405	12,406
*Longibrachiatum*	*T. citrinoviride*	TUCIM 6016	GCF_003025115.1	33.21	533	52.31	9929	9735
	*T. longibrachiatum*	ATCC 18648	GCA_003025155.1	32.23	130	51.25	11,132	10,934
	*T. parareesei*	CBS 125925	GCA_001050175.1	32.07	885	53.47	9062	9062
	*T. reesei*	QM6a	GCF_000167675.1	33.39	77	52.75	9109	9111
*Trichoderma*	*T. asperellum*	CBS 433.97	GCF_003025105.1	37.46	419	47.31	12,775	12,557
	*T. atroviride*	IMI 206040	GCF_000171015.1	36.14	29	49.75	11,809	11,816
	*T. gamsii*	T6085	GCF_001481775.2	37.90	172	48.95	11,171	11,171

**Table 4:** COG functional category distribution of *Trichoderma* spp. Each number in parentheses is relative abundance. The highest value for each COG category is in bold

	*T. asperellum*	*T. atroviride*	*T. citrinoviride*	*T. gamsii*	*T. guizhouense*	*T. harzianum*	*T. lentiforme*	*T. longibrachiatum*	*T. parareesei*	*T. reesei*	*T. simmonsii*	*T. virens*
(A) RNA processing and modification	315 (2.51)	322 (2.73)	323 (3.32)	313 (2.80)	324 (2.88)	328 (2.33)	313 (2.41)	313 (2.86)	316 (3.49)	314 (**3.45**)	**352** (2.54)	329 (2.65)
(B) Chromatin structure and dynamics	204 (1.62)	204 (1.73)	184 (1.89)	205 (1.84)	213 (1.89)	239 (1.70)	230 (1.77)	172 (1.57)	177 (**1.95**)	173 (1.90)	**261** (1.88)	223 (1.80)
(C) Energy production and conversion	387 (3.08)	389 (3.29)	321 (3.30)	393 (3.52)	435 (**3.86**)	454 (3.23)	435 (3.35)	297 (2.72)	307 (3.39)	308 (3.38)	**474** (3.42)	414 (3.34)
(D) Cell cycle control, cell division, chromosome partitioning	171 (1.36)	186 (1.57)	174 (1.79)	169 (1.51)	180 (1.60)	176 (1.25)	168 (1.29)	171 (1.56)	176 (**1.94**)	170 (1.87)	**192** (1.38)	190 (1.53)
(E) Amino acid transport and metabolism	500 (3.98)	478 (4.05)	436 (4.48)	477 (4.27)	549 (**4.88**)	583 (4.15)	554 (4.27)	412 (3.77)	412 (4.55)	411 (4.51)	**641** (4.62)	540 (4.35)
(F) Nucleotide transport and metabolism	125 (1.00)	130 (1.10)	115 (**1.18**)	131 (1.17)	131 (1.16)	145 (1.03)	141 (1.09)	113 (1.03)	113 (1.25)	116 (1.27)	**158** (1.14)	138 (1.11)
(G) Carbohydrate transport and metabolism	642 (5.11)	636 (5.38)	502 (5.16)	628 (5.62)	666 (**5.92**)	691 (4.91)	670 (5.16)	488 (4.46)	492 (5.43)	485 (5.32)	**745** (5.37)	687 (5.54)
(H) Coenzyme transport and metabolism	221 (1.76)	213 (1.80)	194 (1.99)	213 (1.91)	236 (2.10)	240 (1.71)	235 (1.81)	194 (1.77)	191 (**2.11**)	191 (2.10)	**282** (2.03)	230 (1.85)
(I) Lipid transport and metabolism	386 (3.07)	372 (3.15)	337 (3.46)	365 (3.27)	392 (**3.48**)	402 (2.86)	393 (3.03)	310 (2.84)	313 (3.45)	313 (3.44)	**430** (3.10)	410 (3.30)
(J) Translation, ribosomal structure and biogenesis	381 (3.03)	390 (3.30)	375 (3.85)	375 (3.36)	398 (3.54)	402 (2.86)	387 (2.98)	360 (3.29)	363 (**4.01**)	361 (3.96)	**434** (3.13)	397 (3.20)
(K) Transcription	363 (2.89)	362 (3.06)	323 (3.32)	357 (3.20)	377 (3.35)	403 (2.87)	381 (2.94)	318 (2.91)	312 (**3.44**)	307 (3.37)	**462** (3.33)	373 (3.01)
(L) Replication, recombination and repair	255 (2.03)	265 (2.24)	241 (2.48)	265 (2.37)	268 (2.38)	288 (2.05)	284 (2.19)	232 (2.12)	234 (**2.58**)	222 (2.44)	**325** (2.34)	279 (2.25)
(M) Cell wall/membrane/envelope biogenesis	142 (1.13)	162 (1.37)	96 (0.99)	150 (1.34)	156 (**1.39**)	164 (1.17)	168 (1.29)	97 (0.89)	109 (1.20)	93 (1.02)	**182** (1.31)	169 (1.36)
(N) Cell motility	5 (0.04)	5 (0.04)	5 (0.05)	5 (0.04)	5 (0.04)	5 (0.04)	5 (0.04)	5 (0.05)	5 (**0.06**)	5 (0.05)	**6** (0.04)	5 (0.04)
(O) Posttranslational modification, protein turnover, chaperones	610 (4.86)	634 (5.37)	560 (5.75)	619 (5.54)	634 (5.63)	650 (4.62)	612 (4.72)	548 (5.01)	548 (6.05)	536 (**5.88**)	**713** (5.14)	634 (5.11)
(P) Inorganic ion transport and metabolism	267 (2.13)	256 (2.17)	241 (2.48)	248 (2.22)	279 (2.48)	285 (2.03)	285 (2.20)	234 (2.14)	237 (2.62)	237 (**2.60**)	**313** (2.26)	290 (2.34)
(Q) Secondary metabolites biosynthesis, transport and catabolism	550 (4.38)	538 (4.55)	418 (4.29)	544 (4.87)	654 (**5.81**)	681 (4.84)	671 (5.17)	400 (3.66)	400 (4.41)	407 (4.47)	**755** (5.44)	662 (5.34)
(S) Function unknown	2790 (22.22)	2853 (24.15)	2433 (24.99)	2764 (24.74)	2865 (25.46)	3168 (22.52)	3004 (23.15)	2338 (21.38)	2343 (**25.86**)	2305 (25.30)	**3293** (23.73)	3105 (25.03)
(T) Signal transduction mechanisms	358 (2.85)	368 (3.11)	362 (**3.72**)	348 (3.12)	373 (3.31)	388 (2.76)	372 (2.87)	337 (3.08)	342 (3.77)	338 (3.71)	**424** (3.06)	397 (3.20)
(U) Intracellular trafficking, secretion, and vesicular transport	469 (3.73)	475 (4.02)	446 (4.58)	469 (4.20)	497 (4.42)	486 (3.46)	481 (3.71)	415 (3.80)	432 (4.77)	424 (**4.65**)	**561** (4.04)	484 (3.90)
(V) Defense mechanisms	56 (0.45)	68 (0.58)	41 (0.42)	71 (0.64)	82 (0.73)	**93** (**0.66**)	84 (0.65)	37 (0.34)	38 (0.42)	39 (0.43)	91 (**0.66**)	69 (0.56)
(W) Extracellular structures	7 (**0.06**)	6 (0.05)	5 (0.05)	6 (0.05)	6 (0.05)	**8** (**0.06**)	7 (0.05)	5 (0.05)	5 (**0.06**)	5 (0.05)	**8** (**0.06**)	4 (0.03)
(Y) Nuclear structure	27 (0.22)	26 (0.22)	27 (**0.28**)	27 (0.24)	27 (0.24)	28 (0.20)	27 (0.21)	26 (0.24)	26 (0.29)	26 (0.29)	**32** (0.23)	28 (0.23)
(Z) Cytoskeleton	129 (1.03)	136 (1.15)	121 (1.24)	137 (1.23)	137 (1.22)	141 (1.00)	133 (1.02)	118 (1.08)	121 (**1.34**)	119 (1.31)	**154** (1.11)	148 (1.19)

### Reference genomes

To analyze the genomic similarities and differences between *T. simmonsii* GH-Sj1 and related genomes, we collected 11 previously annotated *Trichoderma* genomes from NCBI: *T. asperellum* CBS 433.97, *T. atroviride* IMI 206040, *T. citrinoviride* TUCIM 6016, *T. gamsii* T6085, *T. guizhouense* NJAU 4742, *T. harzianum* CBS 226.95, *T. lentiforme* CFAM-422, *T. longibrachiatum* ATCC 18648, *T. parareesei* CBS 125925, *T. reesei* QM6a, and *T. virens* Gv29–8. Table 3 shows assembly statistics for *T. simmonsii* and the other 11 species. Compared to *T. simmonsii* GH-Sj1 (~ 40 Mb), assembly lengths of the listed genomes ranged from ~ 32 Mb (*T. parareesei)* to ~ 41 Mb (*T. harzianum*). GC contents ranged from ~ 47% to ~ 53%, and the *T. simmonsii* GC content (~ 48%) belongs to this range. The number of genes ranges from ~ 9 K to ~ 13 K, where *T. simmonsii* had the second highest number of genes and proteins following *T. harzianum*.

### Phylogeny of *T. simmonsii*

**Fig. 3:**
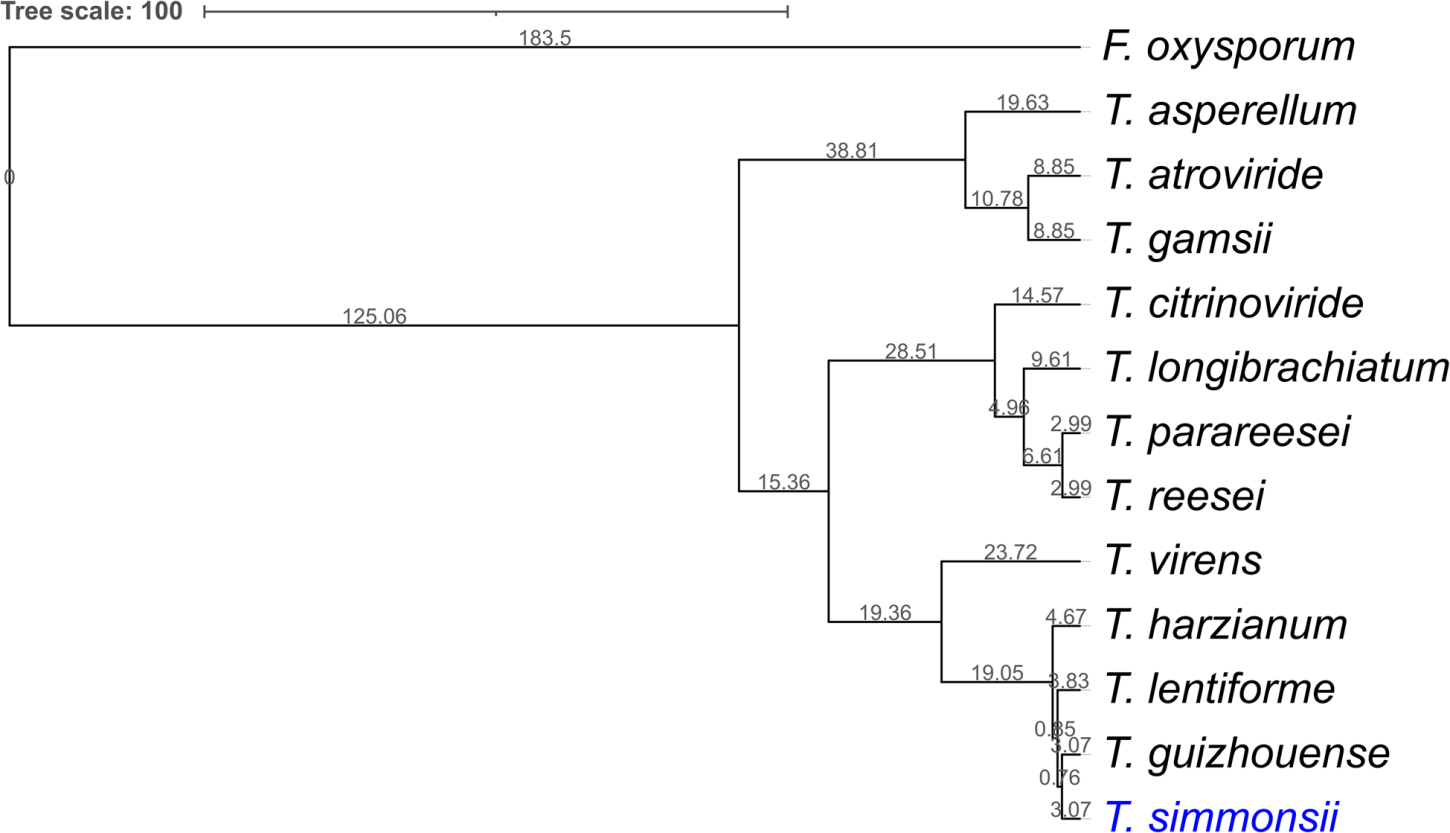
Phylogenetic tree of *Trichoderma simmonsii* GH-Sj1. Orthologous genes were identified by OrthoMCL. Each orthologous group aligned by MUSCLE was concatenated, and a maximum likelihood tree was generated by RAxML. Divergence time was estimated using MEGA with *Fusarium oxysporum* as an out-group. The resulting tree topology was visualized via iTOL online (https://itol.embl.de). The tree scale is in million years ago (MYA). *T. simmonsii* forms monophyletic groups with *Harzianum* clade: *T. guizhouense, T. lentiforme*, and *T. harzianum* along with the *Virens* clade: *T. virens*

After investigating structure and compositions of the reference genomes, we questioned the evolutionary relationships of *T. simmonsii* with other *Trichoderma* spp. To answer the question, we constructed a maximum likelihood phylogenetic tree of *T. simmonsii* using RAxML [33] shown in Fig. 3 using the 11 reference *Trichoderma* species and *F. oxysporum* NRRL-32932 as an out-group. The divergence times of species were calculated via MEGA [34] using the estimated time between *T. harzianum* and *F. oxysporum* (98–269 million years ago (MYA)). The tree topology was concordant with that from a previous study [13]. *T. simmonsii* formed monophyletic groups with the Harzianum clade genomes of *T. guizhouense, T. lentiforme*, and *T. harzianum* along with the *Virens* clade genome of *T. virens*.

### Comparative analyses

Although the genome structure and composition of *T. simmonsii* was not drastically distinct from those of other *Trichoderma* spp., we wondered whether *Trichoderma simmonsii* has a common or unique profile of certain groups of predicted proteins. To address this, various comparative genomic analyses were performed using the funannotate fungal genome analysis suite [29], including comparisons in MEROPS protease families [35], CAZyme families, secreted proteins, and fungal transcription factors. Overall, the distribution of search results from CAZyme, MEROPS, secreted proteins, and fungal transcription factors was similar (Supplementary Figs. 2, 3, 4, 5, 6 and 7) for all *Trichoderma* spp. analyzed. For all protein family searches, protein-coding genes were most abundant in the *T. simmonsii* genome. In addition, we applied the CAFE program [36] to detect rapidly evolving families of *Trichoderma* genomes (Supplementary Fig. 8). *T. simmonsii* had 73 rapidly evolving orthologous gene families, second only to *T. reesei*, which had 94*.* In *T. simmonsii*, 72 were from expanded gene families, and one was from a contracted gene family, whereas only 6 were from expanded families, and 88 were from contracted families in *T. reesei* (Supplementary Table 5). *T. harzianum* had 40 rapidly expanded gene families, second only to *T. simmonsii*. An asparaginase-related gene, H0G86_011897, which included the PFAM domain of PF01112, was detected in the rapidly expanded gene families of *T. simmonsii*. The full list of rapidly evolving protein families in *T. simmonsii* is provided in Supplementary Table 6.

### RNA-sequencing of *T. simmonsii* isolate GH-Sj1

While we screened marine fungi for asparaginase activity, results of the phenol red plate assay indicated that GH-Sj1 produced asparaginase. Because *Trichoderma* spp. are well-known resources for industrial enzyme production [2, 3], we selected GH-Sj1 for transcriptome analysis of genes possibly related to the asparaginase activity.

**Fig. 4:**
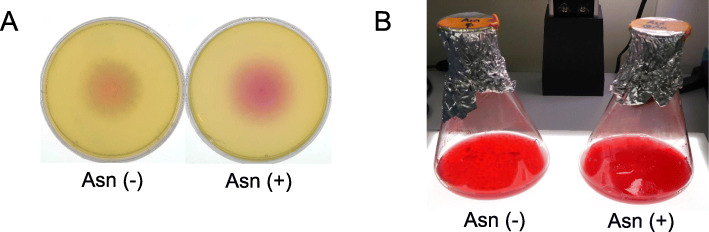
Asparaginase activity of *Trichoderma simmonsii* GH-Sj1. GH-Sj1 inoculated on solid media containing 1% (w/v) asparagine, Asn (+), and no asparagine, Asn (−), as a control. Following incubation at 28 °C for 3 days, the colony color change (pH change due to asparaginase activity) was observed. (B) Before extracting total RNA, GH-Sj1 was inoculated in liquid media containing 1% asparagine (asparaginase-rich conditions, Asn (+)) or no asparagine (non-rich conditions, Asn (−)) and cultured at 25 °C and 200 rpm for 4 days

To perform RNA-sequencing analysis, first, we investigated asparaginase-rich conditions for fungal cultivation based on the results of phenol-red plate assay. GH-Sj1 was cultivated grew on media containing phenol red with or without L-asparagine (Fig. 4A). The color of phenol red is yellow at pH 6.4 or below, becomes red at pH 8.2, and changes into pink above pH 8.2 [37]. When NH_3_ is produced via the hydrolysis of L-asparagine by L-asparaginase, an increase of pH in the cell culture is observed. When grown with L-asparagine, the background color of the GH-Sj1 colony was pink (Fig. 4A). In contrast, on media without L-asparagine, the background color of the GH-Sj1 colony was partially reddish or pink, suggesting a more prominent pH change of the L-asparagine media. This color change was similarly observed in liquid culture of GH-Sj1 for total RNA extraction (Fig. 4B). Therefore, we concluded that addition of L-asparagine to the media resulted in asparaginase-rich conditions of this strain. The media without L-asparagine was used as control conditions.

For transcriptome analysis, both control and experimental samples had two biological replicates: Czp1 and Czp2 for asparaginase non-rich conditions (control); G3 and G4 for asparaginase-rich conditions (experimental samples). RNA sequences for the four samples were generated using the Illumina platform, with sequencing yields of 7,652,661,728 bp from 37,884,464 PE reads, 5,576,060,722 bp from 27,604,261 PE reads, 7,693,022,136 bp from 38,084,268 PE reads, and 6,590,780,250 bp from 32,627,625 PE reads, respectively. After trimming low-quality bases, 35,271,981 PE reads (7,109,693,702 bp), 25,842,265 PE reads (5,209,032,962 bp), 35,517,705 PE reads (7,158,679,180 bp) and 30,568,962 PE reads (6,161,876,011 bp) remained, respectively.

### Putative asparaginase genes in *T. simmonsii* genome

**Fig. 5:**
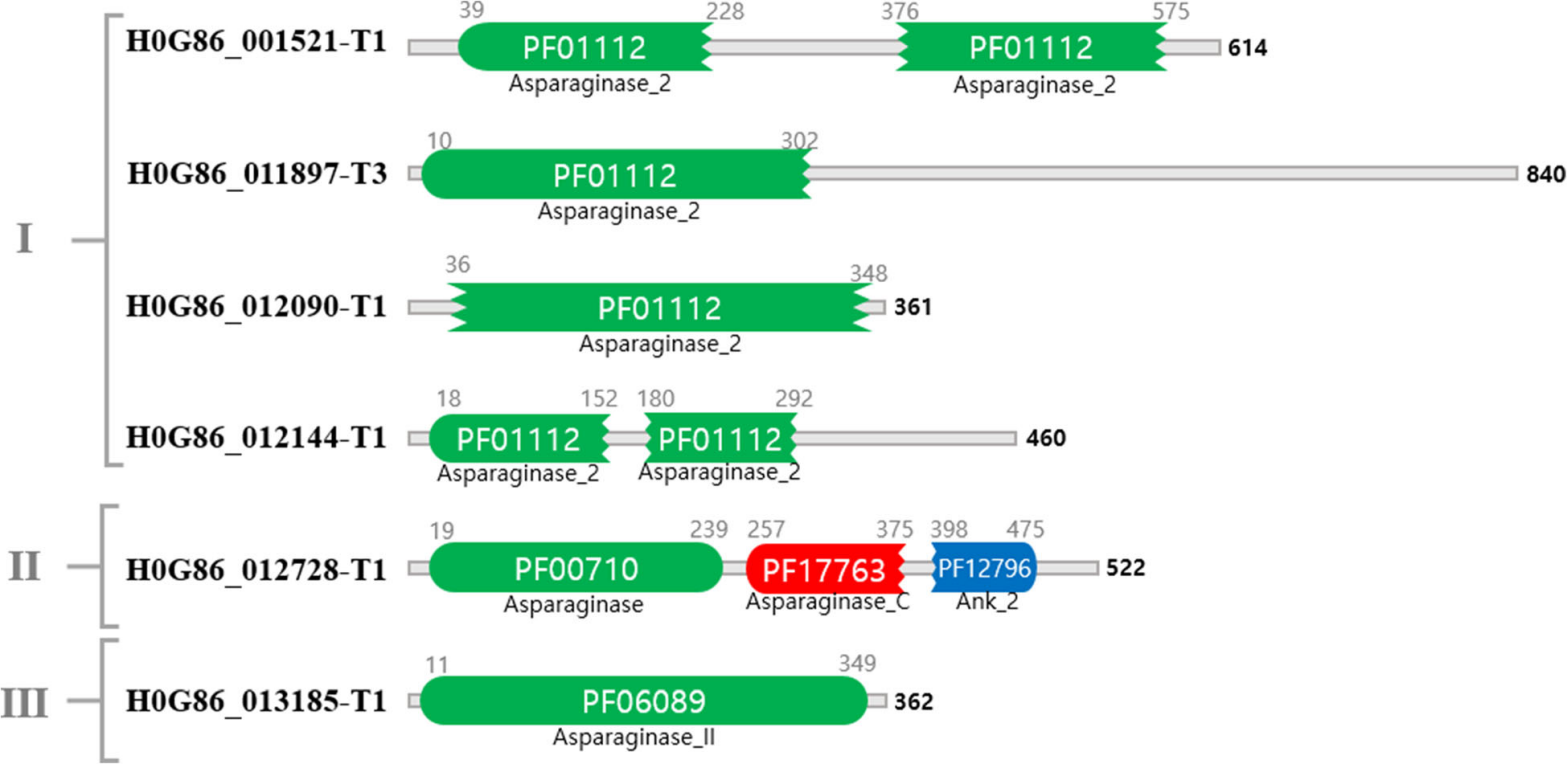
PFAM classes of asparaginases in *Trichoderma simmonsii* GH-Sj1. Asparaginase class I consisted of four genes, which include PF01112. One gene belonged to class II and has two asparaginase domains, PF00710 and PF17763. Class III had one gene, which had PF06089, an L-asparaginase II domain

We performed sequence homology and PFAM asparaginase-related domain searches to uncover genes potentially responsible for the asparaginase activity of GH-Sj1. In total, seven genes in the *T. simmonsii* genome were identified as putative asparaginase-encoding genes: H0G86_001521, H0G86_011897, H0G86_011901, H0G86_012090, H0G86_012144, H0G86_H012728, and H0G86_H013185. The size of amino acids, gene expression levels, and closely related asparaginase-producing fungal species were listed in Table 5. H0G86_011901 does not include a PFAM asparaginase-related domain but was annotated as “putative L-asparaginase” based on the homology search. Six other genes carried at least one PFAM asparaginase domain. Based on the types of PFAM domains(s), 6 asparaginase genes in *T. simmonsii* were classified in 3 categories, as shown in Fig. 5. The majority of genes belonged to Class I, containing the PFAM domain PF01112 (Name: Aspraginase_2; Description: Asparaginase): H0G86_001521, H0G86_011897, H0G86_012090, and H0G86_012144. The E-values of gene pairs measured by BLASTP were between 1.15e-05 and 5.99e-29 (Supplementary Fig. 9). Class II (H0G86_012728) contains two asparaginase PFAM domains, PF00710 (Name: Asparaginase; Description: Asparaginase, N-terminal) and PF17763 (Name: Asparaginase_C; Description: Glutaminase/Asparaginase C-terminal domain). Class III (H0G86_013185) contains PFAM domain PF06089 (Name: Asparaginase_II, Description: L-asparaginase II) [38]. Both H0G86_012728 (Class II) and H0G86_013185 (Class III) were dissimilar among other types of asparaginase, with the lowest E-values being 0.22 and 3.0, respectively (Supplementary Fig. 9).

**Table 5:** Features of putative asparaginase genes of *Trichoderma simmonsii* GH-Sj1

Protein	Amino acids	Log2-fold change	p-value	Adjusted *p*-value	Product	PFAM	Closely related species from BLASTP search	Genbank number	Sequence Identity (%)	E-value
H0G86_011901-T1	571	7.61	1.45E-05	6.99E-05	Putative L-asparaginase	PF07690:Major Facilitator Superfamily	*T. guizhouense*	OPB40701	98.8	0
H0G86_012728-T1	522	3.66	3.30E-16	6.31E-15	Asparaginase	PF00710:Asparaginase, N-terminal; PF17763: Glutaminase/Asparaginase C-terminal	*T. guizhouense*	OPB40005	99.8	0
							*T. harzianum*	KKP02090	98.5	0
H0G86_013185-T1	362	1.70	3.80E-05	1.69E-04	hypothetical protein	PF06089:L-asparaginase II	*T. harzianum*	XP_02477109	95.3	0
							*T. lentiforme*	KAF3066797	95.3	0
							*Neofusicoccum parvum*	EOD46849	61.8	2e-157
							*Aspergillus phoenicis*	RKD36553	61.0	2e-153
							*Akanthomyces lecanii*	QAA74441	60.4	2e-153
H0G86_012144-T1	460	−3.90	8.70E-15	1.46E-13	hypothetical protein	PF01112:Asparaginase	*T. lentiforme*	PKK51684	96.6	0
							*T. harzianum*	KKP00563	93.7	0
H0G86_001521-T1	614	−0.34	0.082	0.15	hypothetical protein	PF01112:Asparaginase	*T. guizhouense*	OPB43587	98.3	0
							*T. harzianum*	XP_024776983	97.2	0
H0G86_011897-T3	840	1.00	0.04	0.08	hypothetical protein	PF01112:Asparaginase	*T. guizhouense*	OPB40705	99.4	
H0G86_012090-T1	361	0.50	0.16	0.26	Asparaginase	PF01112:Asparaginase	*T. guizhouense*	OPB40534	93.4	0
							*T. lentiforme*	KAF3070097	92.5	0
							*T. harzianum*	XP_024770555	92.2	0
							*Colletotrichum incanum*	OHW91971	64.1	7e-150
							*Purpureocillium lilacinum*	XP_018181188	62.00	4e-147

We further investigated the abundances of the three classes of asparaginases in other 11 *Trichoderma* genomes (Supplementary Table 7). The abundance of Class I asparaginase was 2–4 in all the genomes. In case of Class II asparaginase, every genome carried a single copy. Similarly, there was a single copy of Class III asparaginase in all genomes except for *T. citrinoviride* and *T. longibrachiatum* where PFAM domain PF06089 was not found. In addition, we constructed an asparaginase gene tree. Supplementary Fig. 10 shows phylogenetic relationships of the six asparaginase genes in *T. simmonsii* among other *Trichoderma* genomes. As with the genome tree, RAxML was used to generate the maximum likelihood gene tree. Each gene group, the tree topology was similar to genome tree, where *T. simmonsii* formed a monophyletic to *T. guizhouses, T. lentiforme*, *T. harzianum* and *T. virens*. In case of H0G86_011897, only two neighbors existed which from *T. lengtiforme* and *T. guizhouense*.

### Transcriptional profiles of *T. simmonsii* genes in asparaginase-rich conditions

Following identification of the putative asparaginase genes, we investigated the overall transcriptional profile of GH-Sj1 genes in asparaginase-rich conditions relative to the control. After filtering very low-expressed transcripts, 12,165 genes were statistically tested using deseq2. Differentially expressed genes (DEGs) were defined as having an expression change of more than 4-fold under asparaginase-rich conditions relative to non-rich conditions. Among these, 5381 genes were differentially expressed (adjusted *P*-value <= 0.05) between the two conditions. Approximately 75% of the genes (3997 genes out of 5381 genes) exhibited functions that could be predicted, and the rest were related to hypothetical proteins. In total, 1384 genes exhibited a 4-fold or greater transcript level change under asparaginase-rich conditions relative to non-rich conditions. Among these, 591 and 793 genes were up- and down-regulated, respectively. Interestingly, the expression of many genes for functions involving glycoside hydrolase were downregulated. For example, endo-1,3(4)-β-glucanase, endo-1,4-β-xylanase, chitinases, α-galactosidase, and exo-β-1,3-glucanase exhibited decreased transcript levels in asparaginase-rich conditions.

Genes with increased transcript levels under asparaginase-rich conditions compared to non-rich conditions were enriched in the COG categories of (S) Function unknown (33.7%), (E) Amino acid transport and metabolism (8.9%), (Q) Secondary metabolites biosynthesis, transport, and catabolism (7.5%), (G) Carbohydrate transport and metabolism (7.3%), (O) Post-translational modification, protein turnover, chaperones (6.3%), and (C) Energy production and conversion (5.8%). Genes with decreased transcript levels were enriched in (S) Function unknown (36.0%), (G) Carbohydrate transport and metabolism (16.1%), (E) Amino acid transport and metabolism (8.3%), (Q) Secondary metabolites biosynthesis, transport and catabolism (7.2%), (O) Post-translational modification, protein turnover, chaperones (6.6%), and (P) Inorganic ion transport and metabolism (5.0%).

Among the 7 putative asparaginase genes found in our genome analysis of *T. simmonsii*, 3 (H0G86_011901, H0G86_012728, and H0G86_013185) were up-regulated and 1 (H0G86_012144) was down-regulated under asparaginase-rich conditions compared to non-rich conditions. The most dramatic expression change in response to asparaginase-rich conditions was observed in H0G86_011901. The transcript level of H0G86_011901 increased in asparaginase-rich conditions approximately 128-fold, while those of H0G86_012728 and H0G86_013185 increased approximately 12.6- and 3.2-fold, respectively. In contrast, the transcript level of H0G86_012144 decreased approximately 15-fold under asparaginase-rich conditions. The expression of three other genes, including H0G86_001521, H0G86_011897, and H0G86_012090, was not significantly affected by asparaginase-rich conditions.

## Discussion

*Trichoderma* species have been widely used as biocontrol agents and producers of industrial enzymes. In this work, we studied marine-derived *T. simmonsii* strain GH-Sj1 to understand its genomic structure and transcriptional profiles associated with asparaginase production. *T. simmonsii* was previously identified as a new species within the Harzianum clade of *Trichoderma* based on internal transcribed spacer (ITS) and translation elongation factor 1-α (TEF1) sequences [5]. However, relative to other *Trichoderma* species, *T. simmonsii* remains largely unknown.

A complete, telomere-to-telomere, chromosome-level reference genome assembly is a valuable resource and essential for studies on chromosome evolution and lineage-specific adaptation by ensuring that all genomic variants are discovered and studied [39, 40]. With the rapid advance in sequencing technologies, more and more researchers are performing assembly at the chromosome-level [41]. Telomere-to-Telomere (T2T) consortium accomplished a complete human X chromosome assembly and ultimately plans to complete, high quality telomere-to-telomere assemblies from diploid human genomes [40]. In case of larger brewing yeast *Saccharomyces pastorianus* strains, the existing incomplete and highly fragmented genome assembly was improved on chromosome-scale assembly using Oxford Nanopore MinION sequencing [42]. In addition, *Trichoderma reesei* QM6a genome achieved chromosome-level assembly by resequencing using PacBio and Hi-C technologies [27]. In this study, we accomplished telomere-to-telomere assembly of *T. simmonsii* genome using only Oxford Nanopore long reads and Illumina PE reads. We demonstrated that a high quality fungal genome was assembled by comparing and carefully curating outputs from multiple de novo assemblies without reference to existing assemblies or long range contact information from Hi-C.

The assembly quality of *T. simmonsii* genome was 98.7% with a BUSCO value despite a complete genome. Similarly, for complete genomes *S. pastrorianus* and *T. reesei*, BUSCOs were 90.0 and 99.2%, respectively. A chromosome-level genome assembly does not necessarily guarantee a complete BUSCO score. It may be because the assembly is not 100% accurate, but at the same time, the BUSCO value may not be a perfect indicator of genomic quality assessment. As discussed in the paper, the BUSCO assessment fine-tuned the parameters of score and length cutoff to maximize accuracy, but may still fall short of 100% accurate results as both genetic prediction and orthology assignment are challenging tasks, which can be resulted in missing or incorrect BUSCO predictions [43]. This limitation was also observed in the paper where low BUSCO scores could be caused by missing genes in the gene prediction step of the tool [44]. Despite its limitations, without a definitive alternative, BUSCO is still an essential genomic quality assessment tool that includes up-to-date data from many species. Through the identification of orthologs in the genomes of 12 *Trichoderma* species, we demonstrated that *T. simmonsii* was grouped with fungi belonging to the Harzianum clade, in agreement with this previous report.

Overall, the *Harzianum*/*Virens* clade had more rapidly expanded than rapidly contracted gene families, with *T. simmonsii* having the most expanded families within the clade. Gene family expansion is known to provide a significant evolutionary boost wherein selection may play a role in promoting adaptation [45]. Further, rapid gene family expansion is associated with adaptive natural selection in favor of additional copies either in order to increase dosage or the arsenal of molecular weaponry [46]. *T. reesei* and *T. parareesei* of the *Longibrachiatum* clade had similar rapidly evolving gene families to *T. simmonsii*, but the majority were contracted gene families.

For the transcriptome analysis, we focused on asparaginase production of GH-Sj1. Asparaginase is an enzyme used to treat leukemia and to reduce carcinogenic compounds in food [14, 15]. Currently, bacterial asparaginases are mostly used while fungal asparaginases have been poorly characterized, in particular, at the genomic levels. For example, based on data from the National Center for Biotechnology Information (NCBI), the majority of reported L-asparaginase protein sequences originated from bacteria (95.5%). Fungal L-asparaginase sequences account for only approximately 1.7% [15]. L-asparaginase-producing fungal species are represented by those belonging to the *Aspergillus, Penicillium*, *Fusarium*, *Cladosporium*, and *Trichoderma* genera [15]. Moreover, two types of L-asparaginase and related genes have been identified in *Saccharomyces cerevisiae* (baker’s yeast) [47].

Asparaginase activity of GH-Sj1 was examined using media containing a pH indicator phenol red. Although this method has been widely used for screening microorganisms with asparaginase activity [48], it is noteworthy that positive results from this assay require additional analyses (enzyme purification, etc.) to confirm asparaginase production of the microorganisms. Although the degree of color change (pH change) was more significant when asparagine was added to the media, we observed that growth of GH-Sj1 caused color change in the absence of asparagine (Fig. 4). This suggests that other factors in addition to hydrolysis of asparagine by asparaginase could result in pH change in media. Due to this limitation, sometimes media to screen of asparaginase activity could be optimized by changing carbon sources or concentrations of salts and phenol red [49].

Through the results of genome analysis, we identified seven asparaginase-related genes in *T. simmonsii* GH-Sj1. As previously described, H0G86_011901, which was included based solely on strong sequence homology, exhibited the greatest up-regulation under asparaginase-rich conditions. However, without molecular cloning and enzyme purification, it is currently difficult to conclude which gene(s) are responsible for the asparaginase activity of *T. simmonsii* GH-Sj1 under the tested conditions. In order to elucidate the link between genes and asparaginase activity, future studies will include the generation and characterization of null or overexpression mutants for each gene. Furthermore, it should be noted that the transcriptional profiles were investigated at a single time point. Therefore, the expression levels of each gene over the distinct incubation time could give us further insight into its role in asparaginase activity.

## Conclusions

In this study, we sequenced *T. simmonsii* GH-Sj1, which was isolated from sea algae *Saccharina japonica*, using both short and long read platforms. The chromosome-scale *T. simmonsii* genome was obtained through comparing multiple long read assemblers and manual curation. The resulting genome consisted of seven telomere-to-telomere scaffolds with no gaps. The assembled genome was ~ 40 Mb in length and had a GC content of 48.13%. The genome completeness of *T. simmonsii* was ~ 99%. The *T. simmonsii* genome harbored 13,120 protein-coding genes, 176 tRNAs, and an rRNA repeat region, which consisted of seven repeats of the 18S-ITS1–5.8S-ITS2–26S cluster. Through a close homology search and PFAM domain search, seven putative asparaginase-related genes were identified, of which three were up-regulated under asparaginase-rich conditions. To our best of knowledge, this is the first report of the *T. simmonsii* genome, thus representing a valuable resource for the further study of enzymatic activities, including that of asparaginase, as well as comparative studies of fungal genomes.

## Methods

### Sample collection and fungal isolation

Sea algae, *Saccharina japonica*, also called as Kombu, was collected from Gul-Hang Quay at Sacheon, Gyeongsangnamdo Province, Republic of Korea (34.55′43.5″N, 128.03′24.8″E). It was washed with sterile water, cut into about 1-cm segments using sterile scissors, and placed onto potato dextrose agar (PDA; BD) and yeast-mold agar (YM agar; BD) containing 0.01% (w/v) ampicillin and 0.01% (w/v) streptomycin. After incubation at 20 °C for 14 days, fungal colonies were isolated and transferred to fresh PDA until pure spores were obtained. The fungal isolates were stored in 20% glycerol solution at − 80 °C and deposited in the National Marine Biodiversity Institute of Korea (MABIK).

### Morphological characterization of GH-Sj1

Following growth on PDA at 25 °C for 7 days, GH-Sj1 colony morphology was observed and conidia were collected using sterile H_2_O. Conidia and conidiophore morphology were observed using a Leica CTR6000 microscope equipped with a Leica DMC2900 camera (Leica, Germany). Image acquisition and processing were performed using LASV4.5 software (Leica).

### Genomic DNA extraction

Extraction of fungal genomic DNA was performed as previously described [50]. Briefly, fungal isolates were cultured in potato dextrose broth (PDB; BD) at 25 °C, 200 rpm for 3 days. Mycelia were harvested using Miracloth (Millipore), frozen using liquid nitrogen, and ground with a mortar and a pestle. The ground fungal tissue was suspended using lysis buffer followed by addition of phenol: chloroform: isoamyl alcohol (25:24:1) (Sigma, US). After centrifugation at 4 °C and 13,000 rpm for 10 min, the aqueous layer was collected, and genomic DNA was precipitated via the addition of isopropanol. DNA was harvested by centrifugation at 13,000 rpm for 5 min, dried at room temperature, and dissolved with nuclease-free water.

### PCR and fungal identification

For the molecular identification of fungal strain GH-Sj1, polymerase chain reaction (PCR) was performed using primers EF1-728F [17] and TEF1LLErev [18] in order to amplify *tef1α*, which encodes translation elongation factor 1 *α*. PCR running conditions were as follows: 2 min at 94 °C; 35 cycles of 30 s at 94 °C, 30 s at 55 °C, and 1 min at 72 °C, and, finally, 72 °C for 15 min. Purification was then performed using a QIAquick PCR Purification Kit (Qiagen, Germany), and the sequences of PCR products were analyzed by Macrogen (Macrogen, Korea). The obtained sequences of *tef1α* were used to search closely related species in GenBank via BLASTN [51].

### Examination of asparaginase activity

Fungal isolates were cultured on Czapek-Dox broth (CDB, BD) supplemented with 0.1% (w/v) yeast extract, 1% (w/v) L-asparagine monohydrate (Sigma), and 0.005% (w/v) phenol red (Sigma). As a control, the isolates were cultured on the same media without L-asparagine monohydrate. The pH values of all media were adjusted to pH 6.0. To obtain fungal spores, we cultured GH-Sj1 on PDA at 25 °C for 7 days, and collected spores using sterile H_2_O. Five microliters of the spore suspension were inoculated in the center of the phenol red plates. After incubation at 25 °C for 3 days, the color of the medium was observed. Compared to the control plate, the color change from orange to pink in the media containing L-asparaginase was considered to indicate asparaginase activity of the tested strain.

### Total RNA extraction

GH-Sj1 (5 × 10^7^ conidia) was cultured in 100 ml of Czapek-Dox broth supplemented with 0.1% (w/v) yeast extract and 1% (w/v) L-asparagine monohydrate (asparaginase-rich conditions) or without L-asparagine monohydrate (non-rich conditions). Following incubation at 25 °C and 200 rpm for 4 days, mycelia were harvested, flash frozen in liquid nitrogen, and ground in a mortar. Fungal tissue was suspended in 1 ml TRIzol reagent (Invitrogen, US) followed by the addition of 200 μl chloroform (Sigma). After centrifugation at 4 °C and 13,000 rpm for 15 min, the upper layer was transferred to a fresh microcentrifuge tube. RNA was precipitated with 80% ethanol and purified using the RNeasy plant mini kit (Qiagen). This experiment was performed in two biological replicates.

### DNA library construction and sequencing

For Illumina sequencing, a sequencing library with short inserts of 550 bp for paired-end reads were prepared using the Truseq DNA PCR-Free kit as per the manufacturer’s protocol for Illumina (Illumina, San Diego, CA, USA). 2 × 151 bp reads were generated on an Illumina NovaSeq6000 platform (Illumina) at DNALink (Seoul, S. Korea). For long read sequencing, a 1D sequencing library was constructed using Oxford Nanopore Technologies’ standard ligation sequencing kit SQK-LSK109. FLO-MIN106 (R9.4) flow cells were used for sequencing on the GridION X5 platform (Oxford Nanopore Technologies, Oxford, UK) at MABIK.

### RNA library construction and sequencing

Four sets of sequencing libraries for paired-end reads were prepared using the TruSeq Stranded mRNA kit following the manufacturer’s protocol for Illumina (Illumina, San Diego, CA, USA). Products were quantified using the Bioanalyzer 2100 (Agilent, Santa Clara, CA, USA), and 2 × 101 bp reads were generated on an Illumina NovaSeq6000 platform (Illumina) at DNALink.

### Sequence preprocessing

Illumina WGS reads were quality-trimmed using Trimmomatic (v0.36) [52] with the following trimming options “2:30:10 LEADING:3 TRAILING:3 SLIDINGWINDOW:4:15 MINLEN:75.” Similarly, Illumina RNA-sequencing reads were quality-trimmed using the same software with identical options except for MINLEN:50. The base calling of Nanopore read bases was performed using guppy (v3.2.10) from Oxford Nanopore Technologies [53] with default settings, except for high accuracy mode. Prior to the genome assembly, adapter sequences of Nanopore reads were trimmed using PoreChop (v0.2.4) [54].

### Genome assembly and polishing

Nanopore sequencing reads with at least 10 Kb were assembled using Canu (v1.8) [22], Flye (v2.4) [23], Miniasm assembler (v0.3-r179) [24], Shasta (v0.4.0) [25], and Wtdbg2 (v2.3) [26] assemblers, separately. The initial draft assemblies were polished with adapter-trimmed Nanopore reads of all sizes using MarginPolish (v1.3.dev-5,492,204), followed by HELEN [25]. The polished contigs were further polished 5 times with quality-trimmed Illumina PE reads using Pilon (v1.22) [55]. Genome assembly statistics were calculated from QUAST (v4.5) [56], and the completeness of genome assemblies was evaluated using BUSCO (v4.0.6) [28].

### Telomere repeat detection

Tandem repeat finder (trf v4.04) [57] was used to find all tandem repeats on each contig. We then manually inspected the existence of telomere repeats in each terminus of the contig, namely CCCTAA (5′ terminus) and TTAGGG (3′ terminus).

### Scaffolding and assembly correction

Whole-genome pairwise alignment from two different assemblers was performed using chromeister (v0.8) [58], as shown in Supplementary Fig. 11. Based on the assembly results and telomere repeats summarized in Supplementary Table 1, Miniasm was chosen for a baseline draft assembly, as it was in highest continuity, consisting of only nine contigs with N50 of 6.4 Mb and having the greatest support for telomere ends, with five telomere-to-telomere contigs. Seven Miniasm contigs were greater than 1 Mb in length. The remaining two contigs were ~ 28 Kb and ~ 5 Kb in length. The shortest contig (~ 5 Kb) was discarded because it was even shorter than the minimum input read length (10 Kb). The second shortest contig (~ 28 Kb) was identified as the mitochondrial genome after aligning it to mitochondrial genomes of related species. Two overlapping contigs (~ 3.1 Mb and ~ 1.5 Mb), labeled as 5 and 6 in the y-axis in Supplementary Fig. 11, were concatenated because they overlapped more than 50 Kb in the 3′ and 5′ termini. The two contigs were assembled as single contig in Flye and Wtdbg2 assemblers. In addition, the two contigs had a terminus of either TTAGGG (5′ → 3′) or CCCTAA (3′ → 5′) telomere repeats, but not both. The extended scaffold was polished again using the method mentioned above. The longest contig labeled as 1 in the y-axis in the same figure was split to two contigs due to two centromere regions as illustrated in Supplementary Fig. 12. In addition, the scaffold was assembled as two contigs in Wtdbg2 assembly (Supplementary Fig. 11 (b)). The longer part was replaced with the corresponding Wtdbg2 contig after confirming that it contained both 5′ and 3′ telomere sequences. On the other hand, the Wtdbg2 contig corresponding to the shorter region did not have telomere repeats. We performed local assembly using Miniasm with > = 10 Kb Nanopore reads which did not align to other scaffolds by dropping the reads that aligned > = 80% length to the other scaffolds using minimap2. The longest contigs from the new local assembly were recruited after polishing using the methods described earlier. The total number of scaffolds became seven, and the scaffolds were renamed in decreasing order by lengths. Scaffold ends were adjusted by trimming bases with no short read supports using bwa mem (v0.7.15-r1140) [59] and BEDTools (v2.26.0) [60] via genomeCoverageBed.

### Gene prediction and genome annotation

The genome of *T. simmonsii* was annotated structurally and functionally using funannotate (v1.7.4). The step-by-step pipeline usage is well documented in [61]. Briefly, repeat contains were masked using tantan (v13) [62]. After aligning RNA-sequencing data to the genome using HISAT2 (v2.2.0) [63], genome-guided transcriptome assemblies were generated using Trinity (v2.8.5) [64], followed by PASA (v2.4.1) [65]. Since a fungal genome is expected to have high gene density, the --jaccard_clip option was used for this training stage. Multiple gene models were then predicted by 1) aligning protein sequences to UniProt/SwissProt (2020_03) using DIAMOND (v0.9.21) [66] and EXONERATE (v2.4.0) [67]; 2) performing GeneMark-ES (v4.59) [68] in self-training mode; 3) executing Augustus (v3.3.3) [69], GlimmerHMM (v3.0.4) [70] as well as SNAP (v2013_11_29) [71] with PASA hints; and 4) running CodingQuarry (v2.0) [72] with RNA-sequencing alignment. All these outputs were passed into EvidenceModeler (v1.1.1) [73] to select the consensus models among the ab initio and evidence-based gene models. The gene models were filtered based on length cutoff (< 50 bp), spanning gaps, and the existence of transposable elements. The tRNA genes were predicted with tRNAscan-SE (v2.0.5) [74]. Using the funannotate update command, UTRs were added to gene models. Various functional features were assigned, such as Phobius (v1.01) [75] results, antiSMASH (v5) [32], eggnog-mapper (v2.0.1b) [76], InterProScan (v5.50–84.0) [77], HMMer (v3.3) [78] search of PFAM (v33.1) [79], CAZymes (dbCAN v8.0) [31] using HMMer, and the Diamond blastp search of MEROPS (v12.0) [35].

Apart from the funannotate pipeline, protein functions (i.e., product field) were revised in the following manner. Protein sequences were aligned with BLASTP against all dikarya protein sequences in UniProt DB (v2021_03). Matching sequences were kept when the E-value was <= 1.0e-10, percent identity > = 50%, and query coverage in alignment > = 50%. Protein function was taken from the top hit. When more than one protein sequences from the same gene had a different functional description, we manually corrected them to have same functional description.

### Circular genome map

A circular genome map was drawn using ShinyCircos [80]. From outside to inside, the first ring shows the locus of scaffolds. The second and third rings are mapping coverages of Illumina and Nanopore reads. Illumina DNA reads were aligned using bwa mem with default parameters. For Nanopore reads, minimap2 was used with -x map-ont -a options. The resulting bwa and minimap2 BAM files were sorted in genomic locus order, and base coverage was calculated using genomeCoverageBed with -d option. Average coverage in a 1 Kb window was measured, and the log2 of average coverage was used for efficient coverage plotting due to the existence of several very high-coverage regions which made other regions indistinguishable. The fourth ring is the GC content line which was also drawn in the 1 Kb window. The fifth ring is the gene counts in the 100 Kb window plotted as bar charts. The track and names above the title are the loci of asparaginase-related genes.

### Comparative genomics

In order to perform comparative analyses of *T. simmonsii* to 11 reference genomes under equivalent conditions, the reference genomes were re-annotated using funannotate. Funannotate was then used to perform comparative analyses of functional categories such as PFAM, InterProScan, CAZyme, MEROPS, secreted proteins, and fungal transcription factors.

### Orthology and phylogeny

Orthologous protein sequences of *T. simmonsii* and 12 reference genomes were identified using OrthoMCL (v2.0.9) [81]. Orthologous group consisting of single protein sequences across all genomes were aligned using MUSCLE (v3.8.31) [82] with default options. After concatenation of all orthologous groups, gap regions were trimmed using trimAl (v1.4.rev6) [83] with the -phylip -gappyout option. A maximum likelihood tree was generated using RAxML (v8.2.10) [33] with the following options: -m PROTGAMMAJTT -× 12,345 -p 12345 -N 100 -f a -T 8. Divergence times in the tree were estimated using MEGA (v7.0) [34] with -O *F. oxysporum* -C *‘T. harzianum F. oxysporum* 98269’ options. *F. oxysporum* was assigned as an outgroup, and the time interval between *F. oxysporum* and *T. harzianum* (98, 269) taken from TimeTree [84] was used for branching calculation. CAFE (v4.2.1) [36] was used to identify rapidly evolving families by inputting OrthoMCL output and divergence time estimated from MEGA.

### DEGs

Four RNA read sets (two controls and two experiments) were aligned against the transcriptome using Salmon aligner (v1.4.0) [85]: i.e. salmon quant with -l A --validateMappings options. The mapping results were loaded to deseq2 [86] using tximport function on R (v3.6.0) [87]. Genes expressed at a very low level were removed when the maximum mapping count of each group’s median value was below 10. Subsequent DEG analyses were performed as per the deseq2 manual. Genes whose transcript levels changed 4-fold or greater were included as DEGs.

## Supplementary Information

**Additional file 1.**

**Additional file 2.**

**Additional file 3.**

## Data Availability

This project has been deposited in NCBI under BioProject accession PRJNA645793. BioSample accessions for WGS and four RNA samples are SAMN15516371, SAMN19276232, SAMN19276234, SAMN19276235, and SAMN19276236. Raw sequence reads have been deposited in the SRA under accession number SRR14597877 through SRR14597884. The locus tag of *T. simmonsii* is H0G86 and the NCBI accession numbers for seven scaffolds are CP075864 through CP075870. All data is publicly available from NCBI and can be accessed at https://www.ncbi.nlm.nih.gov/bioproject/PRJNA645793.

## Abbreviations

bp: base pair
CAZyme: Carbohydrate-active enzymes
COG: Clusters of orthologous groups of proteins
DEG: Differentially expressed gene
Gb: Gigabase
GO: Gene ontology
ITS: Internal transcribed spacer
Kb: Kilobase
MABIK: National Marine Biodiversity Institute of Korea
Mb: Megabase
MYA: Million years ago
NCBI: National Center for Biotechnology Information
PCR: Polymerase chain reaction
PE: Paired-end
RNA: Ribonucleic acid
rRNA: ribosomal ribonucleic acid
tRNA: transfer ribonucleic acid
