# Supplementary material for: Telomere-to-telomere genome assembly of asparaginase-producing Trichoderma simmonsii
Source: BMC Genomics. 2021 Nov 17;22:830. doi: 10.1186/s12864-021-08162-4 (PMC8600724; doi:10.1186/s12864-021-08162-4)
Supplement: Supplementary file 1 — Additional file 1. [file 12864_2021_8162_MOESM1_ESM.docx]

| **Assembly** | **Contigs** | **Length (bp)** | **N50 length (bp)** | **L50** | **BUSCO (%)** | **GC (%)** | **Contigs**  **>=100Kbp** | **Telomere (3’ end)** | **Telomere (5’ end)** | **Telomere (both)** |
| --- | --- | --- | --- | --- | --- | --- | --- | --- | --- | --- |
| **Canu** | 20 | 40,232,384 | 2,973,630 | 6 | 98.8 | 48.04 | 14 | **6** | **6** | 0 |
| **Flye** | 13 | 40,119,492 | **6,453,654** | **3** | 98.8 | 48.13 | 8 | 4 | 2 | 1 |
| **Miniasm** | **9** | 40,142,533 | 6,451,180 | **3** | 98.7 | 48.15 | **7** | **6** | **6** | **5** |
| **Shasta** | 105 | 40,034,435 | 3,757,648 | 5 | 98.8 | 48.27 | 14 | 1 | 0 | 0 |
| **Wtdbg2** | 12 | 40,097,630 | 6,445,388 | **3** | **98.9** | 48.10 | **7** | 2 | 1 | 1 |

**Supplementary Table 1. Initial draft assembly comparison.** The best value in each category is written in bold.

| **Scaffold 1 (7,849,262 bp)** | |
| --- | --- |
| 1 | CTAA CCCTAA CCCTAA CCCTAA CCCTAA CCCTAA CCCTAA CCCTAA CCCTAA CCCTAA CCCTAA CCCTAA CCCTAA |
| repeat1 | CTAA CCCTAA CCCTAA CCCTAA CCCTAA CCCTAA CCCTAA CCCTAA CCCTAA CCCTAA CCCTAA CCCTAA CCCTAA |
| 7849186 | TTAGGG TTAGGG TTAGGG TTAGGG TTAGGG TTAGGG TTAGGG TTAGGG TTAGGG TTAGGG TTAGGG TTAGGG TTAGG- |
| repeat2 | TTAGGG TTAGGG TTAGGG TTAGGG TTAGGG TTAGGG TTAGGG TTAGGG TTAGGG TTAGGG TTAGGG TTAGGG TTAGGG |
| **Scaffold 2 (7,394,996 bp)** | |
| 1 | CCAA CCCTAA CCCCCTAA CCCTAA CCCTAA CCCTAA CCCTAA CCCTAA CCCTAA CCCTAA CCCTAA CCCTAA CCCTAA |
| repeat1 | CTAA CCCTAA --CCCTAA CCCTAA CCCTAA CCCTAA CCCTAA CCCTAA CCCTAA CCCTAA CCCTAA CCCTAA CCCTAA |
| 7394909 | TTAGGG TTAGGG TTAGGG TTAGGG TTAGGG TTAGGG TTAGGG TTAGGG TTAGGG TTAGGG TTAGGG TTAGGG TTAGGG |
| repeat2 | TTAGGG TTAGGG TTAGGG TTAGGG TTAGGG TTAGGG TTAGGG TTAGGG TTAGGG TTAGGG TTAGGG TTAGGG TTAGGG |
| 7394987 | TTAGGG TTGG |
| repeat2 | TTAGGG TTAG |
| **Scaffold 3 (6,451,197 bp)** | |
| 1 | T CCCTAA CCCTAA CCCTAA CCCTAA CCCTAA CCCTAA CCCTAA CCCTAA CCCTAA CCCTAA CCCTAA CCCTAA CCCTAA |
| repeat1 | A CCCTAA CCCTAA CCCTAA CCCTAA CCCTAA CCCTAA CCCTAA CCCTAA CCCTAA CCCTAA CCCTAA CCCTAA CCCTAA |
| 80 | CCCTAA |
| repeat1 | CCCTAA |
| 6451111 | TTAGGG TTAGGG TTAGGG TTAGGG TTAGGG TTAGGG TTAGGG TTAGGG TTAGGG TTAGGG TTAGGG TTAGGG TTAGGG |
| repeat2 | TTAGGG TTAGGG TTAGGG TTAGGG TTAGGG TTAGGG TTAGGG TTAGGG TTAGGG TTAGGG TTAGGG TTAGGG TTAGGG |
| 6451189 | TT-GGG TTAG |
| repeat2 | TTAGGG TTAG |
| **Scaffold 4 (4,887,860 bp)** | |
| 1 | AA CC-TAA CCCTAA CCCTAA CCCTAA CCCTAA CCCTAA CCCTAA CCCTAA CCCTAA CCCTAA CCCTAA CCCTAA |
| repeat1 | AA CCCTAA CCCTAA CCCTAA CCCTAA CCCTAA CCCTAA CCCTAA CCCTAA CCCTAA CCCTAA CCCTAA CCCTAA |
| 4887771 | ATAGGG TTAGGG TTAGGG TTAGGG TTAGGG TTAGGG TTAGGG TTAGGG TTAGGG TTAGGG TTAGGG TTAGGG TTAGGG TTAGGG |
| repeat2 | TTAGGG TTAGGG TTAGGG TTAGGG TTAGGG TTAGGG TTAGGG TTAGGG TTAGGG TTAGGG TTAGGG TTAGGG TTAGGG TTAGGG |
| 4887855 | TTAGGG |
| repeat2 | TTAGGG |
| **Scaffold 5 (4,639,282 bp)** | |
| 1 | AA CCC-AA CCCTAA CCC-AA CCC-AA CCC-AA CCC-AA CCCTAA CCCTAA CCCTAA CCCTAA CCCTAA CCCTAA |
| repeat1 | AA CCCTAA CCCTAA CCCTAA CCCTAA CCCTAA CCCTAA CCCTAA CCCTAA CCCTAA CCCTAA CCCTAA CCCTAA |
| 4639213 | TTAGGG TTAGGG TTAGGG TTAGGG TTAGGG TTAGGG TTAGGG TTAGGG TTAGGG TTAGGG TTA-GG TTAGG |
| repeat2 | TTAGGG TTAGGG TTAGGG TTAGGG TTAGGG TTAGGG TTAGGG TTAGGG TTAGGG TTAGGG TTAGGG TTAGG |
| **Scaffold 6 (4,539,006 bp)** | |
| 1 | TAA CCCTAA CCCTAA CCCTAA CCCTAA CCCTAA CCCTAA CCCTAA CCCTAA CCCTAA CCCTAA CCCTAA CCCTAA |
| repeat1 | TAA CCCTAA CCCTAA CCCTAA CCCTAA CCCTAA CCCTAA CCCTAA CCCTAA CCCTAA CCCTAA CCCTAA CCCTAA |
| 76 | CCCTAA ACCTAA CCCTAA CCCT-A CCCTAA CTTTCT |
| repeat2 | CCCTAA CCCTAA CCCTAA CCCTAA CCCTAA CCCTAA |
| 4538917 | TTAGGG TTAGGG TTAGGG TTAGGG TTAGGG TTAGGG TTAGGG TTAGGG TTAGGG TTAGGG TTAGGG TTAGGG TTAGGG |
| repeat2 | TTAGGG TTAGGG TTAGGG TTAGGG TTAGGG TTAGGG TTAGGG TTAGGG TTAGGG TTAGGG TTAGGG TTAGGG TTAGGG |
| 4538995 | TTAGGG TTAGGG |
| repeat2 | TTAGGG TTAGGG |
| **Scaffold 7 (4,316,782 bp)** | |
| repeat1 | A CCC-AA CCCTAA CCCTAA CCCTAA CCCTAA CCCTAA CCCTAA CCCTAA CCCTAA CCCTAA CCCTAA CCCTAA CCCTA- |
| 1 | A CCCTAA CCCTAA CCCTAA CCCTAA CCCTAA CCCTAA CCCTAA CCCTAA CCCTAA CCCTAA CCCTAA CCCTAA CCCTAA |
| 4316684 | ATAGGG TTAGGG TTAGGG TTAGGG TTAGGG TTAGGG TTAGGG TTAGGG TTAGGG TTAGGG TTAGGG TTAGGG TTAGGG |
| repeat2 | TTAGGG TTAGGG TTAGGG TTAGGG TTAGGG TTAGGG TTAGGG TTAGGG TTAGGG TTAGGG TTAGGG TTAGGG TTAGGG |
| 4316762 | TTAGGG TTAGGG TTAGGG TTA |
| repeat2 | TTAGGG TTAGGG TTAGGG TTA |

**Supplementary Table 2. Alignment of telomere repeats.** For each scaffold, 5’ terminus was aligned to CCCTAA (repeat1) and 3’ end region was aligned to TTAGGG (repeat2). Mismatches and gaps in an alignments were shaded in grey. Insertion and deletion gaps were marked with ‘-’. For each telomere repeat, single mismatch and up to two consecutive gaps were allowed. CCCTAA and TTAGGG repeat regions in scaffolds were highlighted in red and blue colors, respectively. The occurrences of CCCTAA repeats in seven scaffolds were 13, 12, 14 12, 12, 17, and 13, respectively. Likewise, TTAGGG repeat counts were 13, 14, 15, 15, 12, 15, and 16, respectively.

| **tRNA** | **Count** |
| --- | --- |
| **Ala** | 14 |
| **Arg** | 13 |
| **Asn** | 6 |
| **Asp** | 9 |
| **Cys** | 2 |
| **Gln** | 7 |
| **Glu** | 12 |
| **Gly** | 13 |
| **His** | 5 |
| **Ile** | 9 |
| **Leu** | 14 |
| **Lys** | 9 |
| **Met** | 4 |
| **Phe** | 3 |
| **Pro** | 10 |
| **Ser** | 14 |
| **Thr** | 11 |
| **Trp** | 3 |
| **Tyr** | 4 |
| **Val** | 11 |
| **iMet** | 3 |

Supplementary Table 3. tRNA gene occurrences

| **antiSMASH** | **Scaffold** | **start** | **end** |
| --- | --- | --- | --- |
| **NRPS** | 2 | 2,694,279 | 2,735,752 |
|  | 4 | 509,093 | 564,810 |
|  |  | 4,842,287 | 4,887,860 |
|  | 6 | 2,232,806 | 2,275,358 |
|  | 7 | 469,482 | 518,109 |
| **NRPS;indole** | 2 | 7,315,195 | 7,380,147 |
| **NRPS;NRPS-like;T1PKS** | 7 | 181,630 | 274,606 |
| **NRPS;T1PKS** | 1 | 147,288 | 196,779 |
|  |  | 391,175 | 479,827 |
|  | 2 | 1,374,426 | 1,456,107 |
|  | 3 | 6,062,224 | 6,173,320 |
|  | 5 | 77472 | 130,158 |
|  | 6 | 3,054,156 | 3,156,871 |
|  | 7 | 290,697 | 338,478 |
| **NRPS;T1PKS;betalactone** | 2 | 598,945 | 652,214 |
| **NRPS-like** | 2 | 419,505 | 456,817 |
|  | 3 | 2,051,705 | 2,093,010 |
|  |  | 2,812,648 | 2,848,429 |
|  |  | 4,311,386 | 4,347,591 |
|  | 4 | 4,672,397 | 4,707,976 |
|  |  | 4,750,622 | 4,792,823 |
|  | 6 | 2,685,322 | 2,731,509 |
|  | 7 | 592,231 | 635,456 |
| **NRPS-like;T1PKS** | 6 | 3,947,755 | 4,021,772 |
| **T1PKS** | 1 | 68,228 | 109,883 |
|  |  | 530,553 | 566,701 |
|  |  | 1,142,601 | 1,182,511 |
|  |  | 6,521,485 | 6,567,489 |
|  |  | 7,126,116 | 7,174,332 |
|  | 2 | 4,077,948 | 4,138,875 |
|  |  | 4,686,624 | 4,726,392 |
|  |  | 4,777,398 | 4,819,593 |
|  |  | 6,573,425 | 6,621,281 |
|  | 3 | 4,793,490 | 4,837,672 |
|  |  | 5,453,645 | 5,490,609 |
|  | 4 | 665,714 | 708,559 |
|  |  | 905,819 | 950,186 |
|  |  | 1,632,766 | 1,679,175 |
|  | 6 | 119,212 | 162,029 |
|  |  | 4,290,927 | 4,338,195 |
|  | 7 | 1,537,473 | 1,576,889 |
|  |  | 1,702,701 | 1,750,693 |
| **terpene** | 2 | 328,069 | 345,980 |
|  |  | 5,681,443 | 5,697,261 |
|  |  | 6,034,028 | 6,055,377 |
|  | 3 | 2,159,743 | 2,178,236 |
|  |  | 2,667,396 | 2,688,571 |
|  | 6 | 1,687,854 | 1,708,794 |
|  | 7 | 398,862 | 420,157 |

Supplementary Table 4. antiSMASH result.

| **Species** | **Expanded families** | **Genes gained** | **Contracted families** | **Genes lost** | **No change** |
| --- | --- | --- | --- | --- | --- |
| ***F. oxysporum*** | 1242 (4) | 2734 | 5122 (0) | 5137 | 7359 |
| ***T. asperellum*** | 126 (6) | 153 | 1118 (0) | 1121 | 12479 |
| ***T. atroviride*** | 110 (3) | 121 | 513 (0) | 513 | 13100 |
| ***T. citrinoviride*** | 166 (0) | 169 | 619 (0) | 619 | 12938 |
| ***T. gamsii*** | 162 (5) | 177 | 1157 (6) | 1172 | 12404 |
| ***T. guizhouense*** | 86 (15) | 92 | 1846 (7) | 1856 | 11791 |
| ***T. harzianum*** | 143 (40) | 191 | 807 (4) | 814 | 12773 |
| ***T. lentiforme*** | 76 (16) | 79 | 1157 (3) | 1161 | 12490 |
| ***T. longibrachiatum*** | 22 (0) | 22 | 559 (2) | 562 | 13142 |
| ***T. parareesei*** | 78 (12) | 79 | 479 (60) | 480 | 13166 |
| ***T. reesei*** | 12 (6) | 12 | 435 (88) | 437 | 13276 |
| ***T. simmonsii*** | 717 (72) | 851 | 860 (1) | 861 | 12146 |
| ***T. virens*** | 184 (9) | 224 | 1940 (1) | 1941 | 11599 |

**Supplementary Table 5. Gene family expansion and contractions.** The numbers in the parenthesis are the count of rapidly changing families.

| **Asparaginase Type** | | **1** | **2** | | **3** |
| --- | --- | --- | --- | --- | --- |
| **PFAM** | **Accession** | PF01112 | PF00710 | PF17763 | PF06089 |
|  | **Name** | Asparaginase_2 | Asparaginase | Asparaginase_C | Asparaginase_II |
|  | **Description** | Asparaginase | Asparaginase, N-terminal | Glutaminase/Asparaginase C-terminal domain | L-asparaginase II |
| **Genome** | ***T. asperellum*** | 3 | 1 | | 1 |
|  | ***T. atroviride*** | 3 | 1 | | 1 |
|  | ***T. citrinoviride*** | 3 | 1 | | 0 |
|  | ***T. gamsii*** | 2 | 1 | | 1 |
|  | ***T. guizhouense*** | 4 | 1 | | 1 |
|  | ***T. harzianum*** | 3 | 1 | | 1 |
|  | ***T. lentiforme*** | 4 | 1 | | 1 |
|  | ***T. longibrachiatum*** | 3 | 1 | | 0 |
|  | ***T. parareesei*** | 3 | 1 | | 1 |
|  | ***T. reesei*** | 3 | 1 | | 1 |
|  | ***T. simmonsii*** | 4 | 1 | | 1 |
|  | ***T. virens*** | 3 | 1 | | 1 |

Supplementary Table 7. Asparaginases of *Trichoderma* genomes
