# Supplementary material for: Telomere-to-telomere genome assembly of asparaginase-producing Trichoderma simmonsii
Source: BMC Genomics. 2021 Nov 17;22:830. doi: 10.1186/s12864-021-08162-4 (PMC8600724; doi:10.1186/s12864-021-08162-4)
Supplement: Supplementary file 2 — Additional file 2. [file 12864_2021_8162_MOESM2_ESM.docx]

**Supplementary Figure 1. COG categories in *T. simmonsii* GH-Sj1**

The top COG category found in *T. simmonsii* GH-Sj1 genomes were annotated as (S) Function unknown, where 3,293 proteins were in this category. The next top 5 COG categories are (Q) Secondary metabolites biosynthesis, transport and catabolism, (G) Carbohydrate transport and metabolism, (O) Posttranslational modification, protein turnover, chaperones, (E) Amino acid transport and metabolism, and (U) Intracellular trafficking, secretion, and vesicular transport.


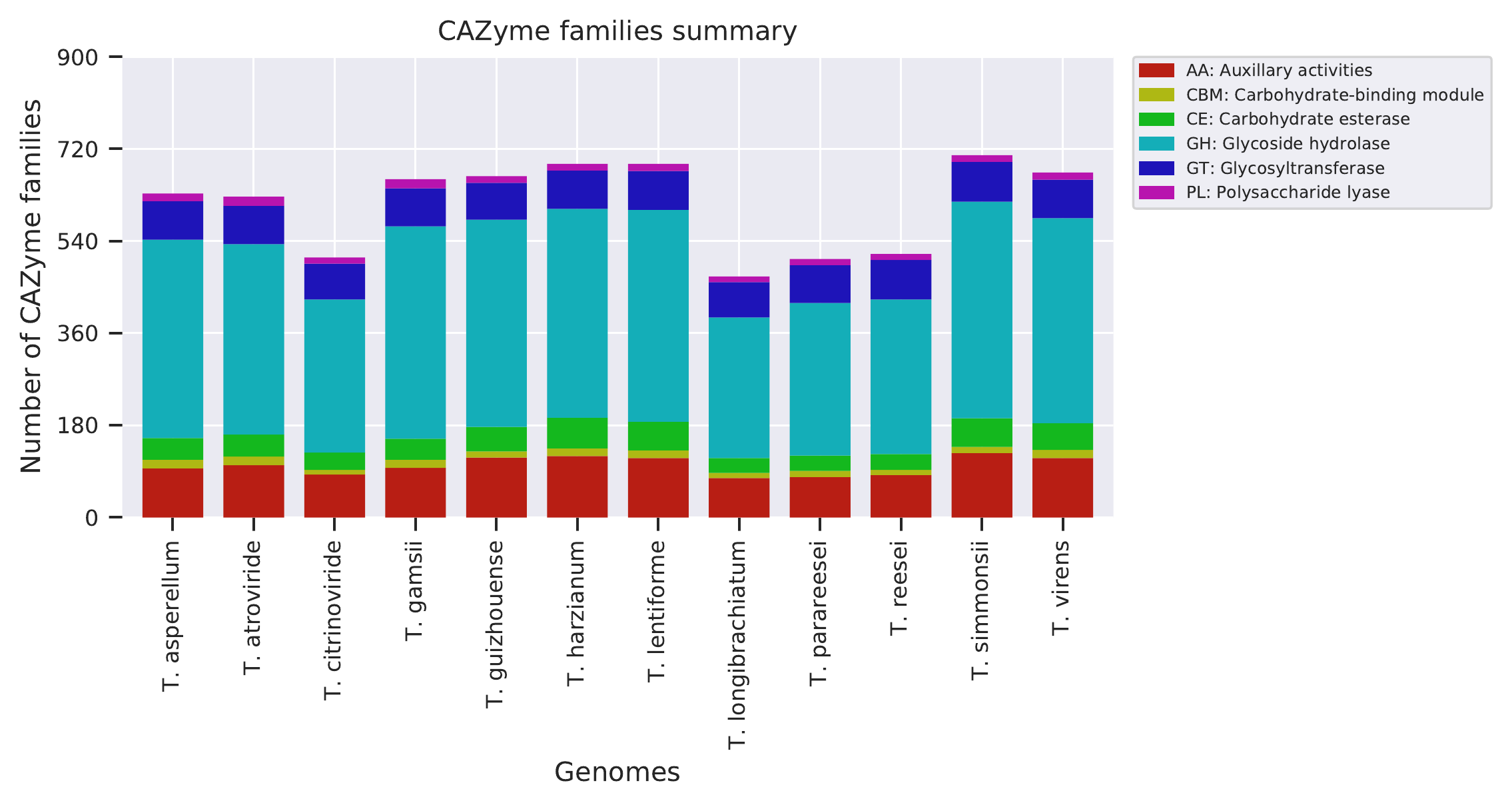


**Supplementary Figure 2. CAZyme summary of *Trichoderma* genomes.**

The overall CAZyme family’s profiles are similar among the 12 genomes. *T. simmonsii* has the most cumulative occurrences. *T. simmonsii* genome has the CAZyme family matches in the following orders: 423 glycoside hydrolases, 126 auxiliary activities, 78 glycosyltransferases, 56 carbohydrate esterases, 13 polysaccaride lyases, and 12 carbohydrate-binding modules.


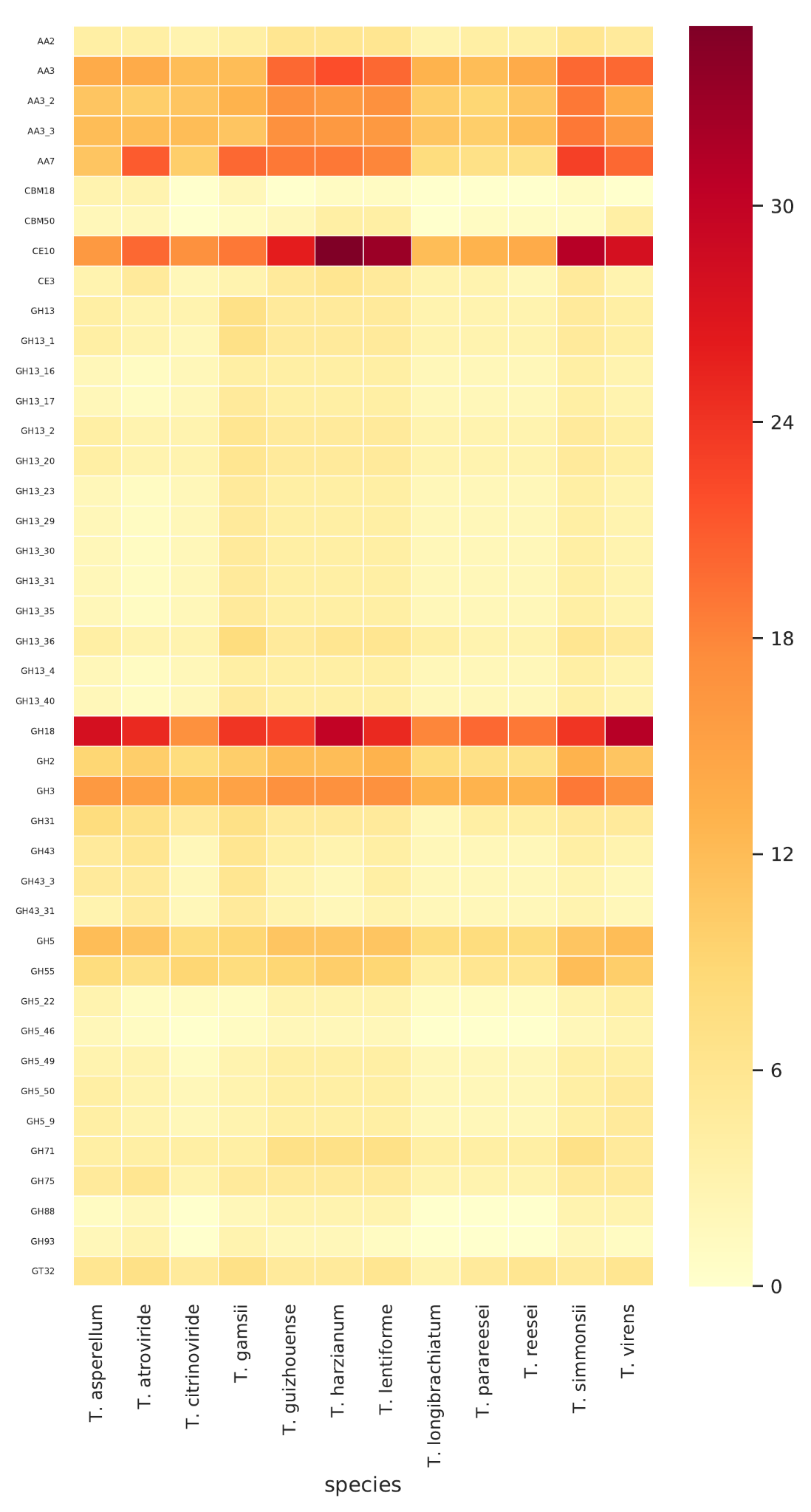


**Supplementary Figure 3. CAZyme abundances of *Trichoderma* genomes.**

The abundance levels of CAZyme matches in 12 *Trichoderma* genomes are similar. The following CAZyme classes has at least double digit abundances in *T. simmonsii*: 31 enzymes for CE10: , 24 for GH18, 23 for AA7, 20 for AA3, 19 for AA3_2, 19 for AA3_3, 19 for GH3, 16 for GH16, 13 for GH2, 12 for GH55, and 11 enzymes for GH5


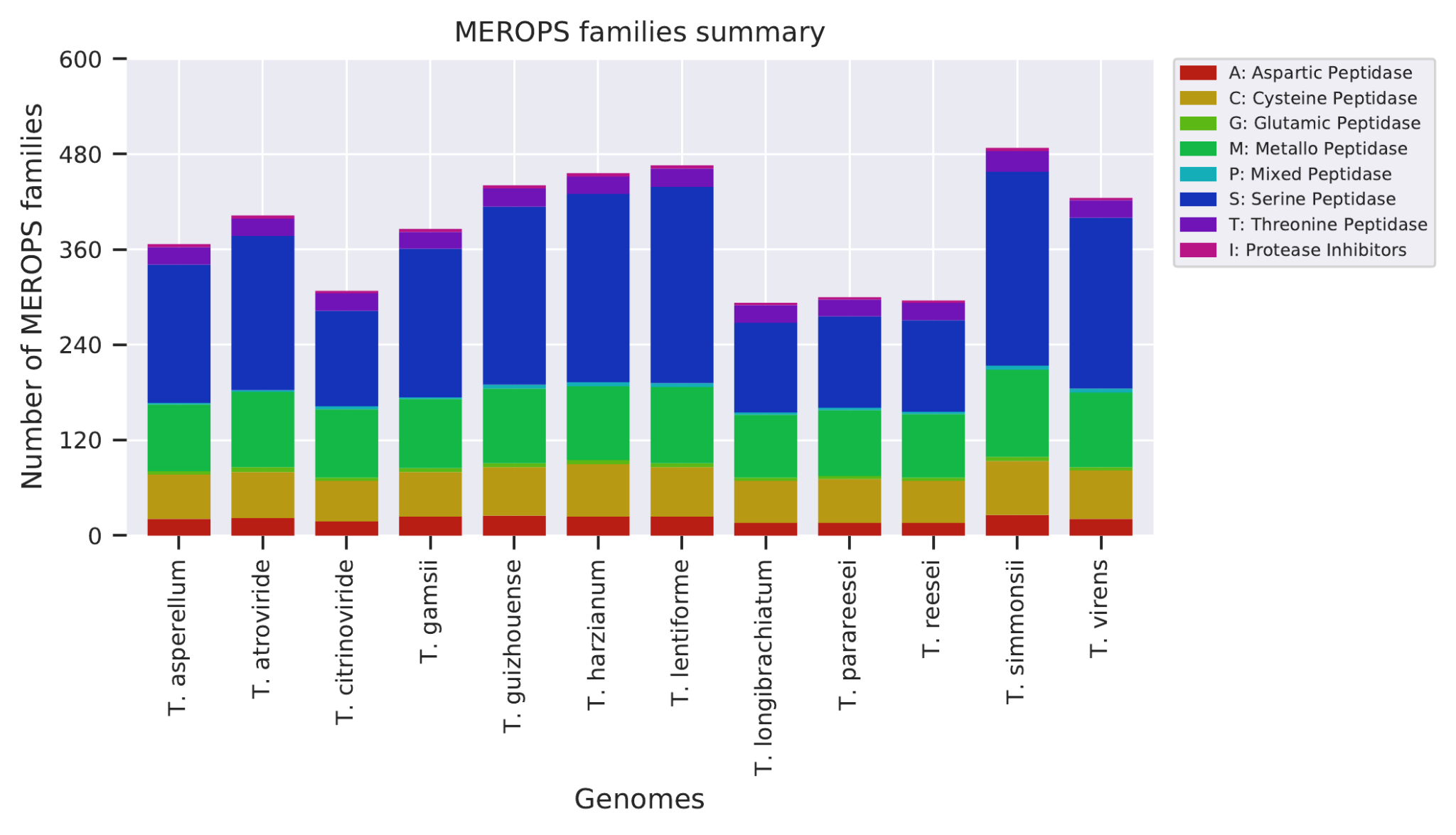


**Supplementary Figure 4. MEROPS summary of *Trichoderma* genomes.**

Overall protease profiles across *Trichoderma* genomes are similar. *T. simmonsii* has most occurrences in all categories. Specifically, *T. simmonsii* has 244 serine peptidases, 110 metallo peptidases, 68 cysteine peptidase, 26 aspartic peptidases, 26 threonine peptidases, 5 glutamic peptidases, 5 mixed peptidases and 4 protease inhibitors.


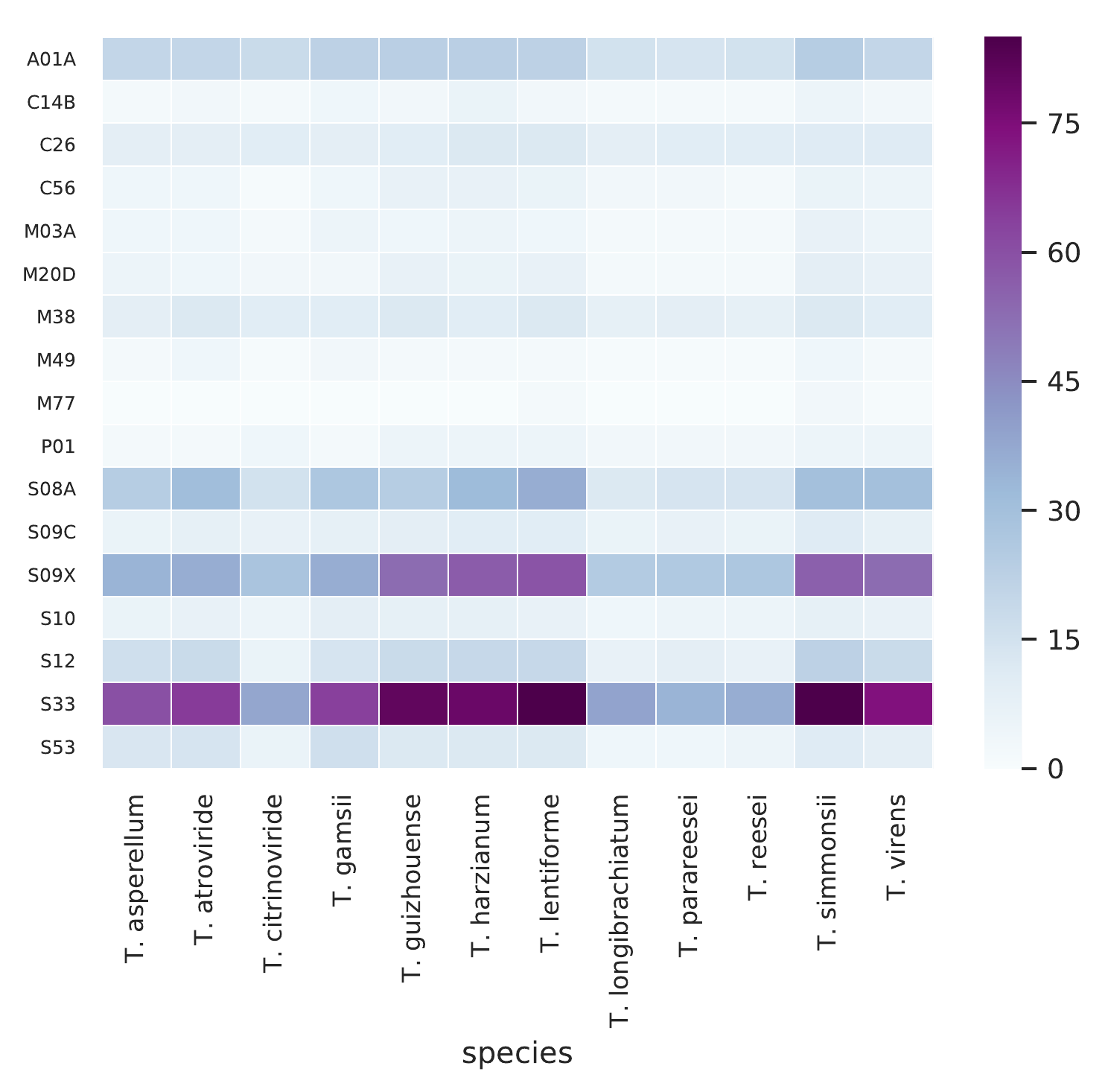


**Supplementary Figure 5. MEROPS abundances of *Trichoderma* genomes.**

The abundance levels of MEROPS identifiers in 12 *Trichoderma* genomes are similar. For *T. simmonsii*, the following MEROPS entries have double digit: 85 for S33: prolyl aminopeptidase, 56 for S09X: Peptidase family S9 (prolyl oligopeptidase family), 30 for S08A: Peptidase family S8 (subtilisin family), 24 for A01A: Peptidase family A1 (pepsin family), 22 for S12: Peptidase family S12 (D-Ala-D-Ala carboxypeptidase B family), 16 for C19: Peptidase family C19 (ubiquitin-specific protease family), 14 for T01A: Peptidase family T1 (proteasome family), 12 for M38: Peptidase family M38 (beta-aspartyl dipeptidase family), 11 for C26: Peptidase family C26 (gamma-glutamyl hydrolase family), 11 for S09C: Peptidase family S9 (prolyl oligopeptidase family), and 11 for S53: sedolisin.


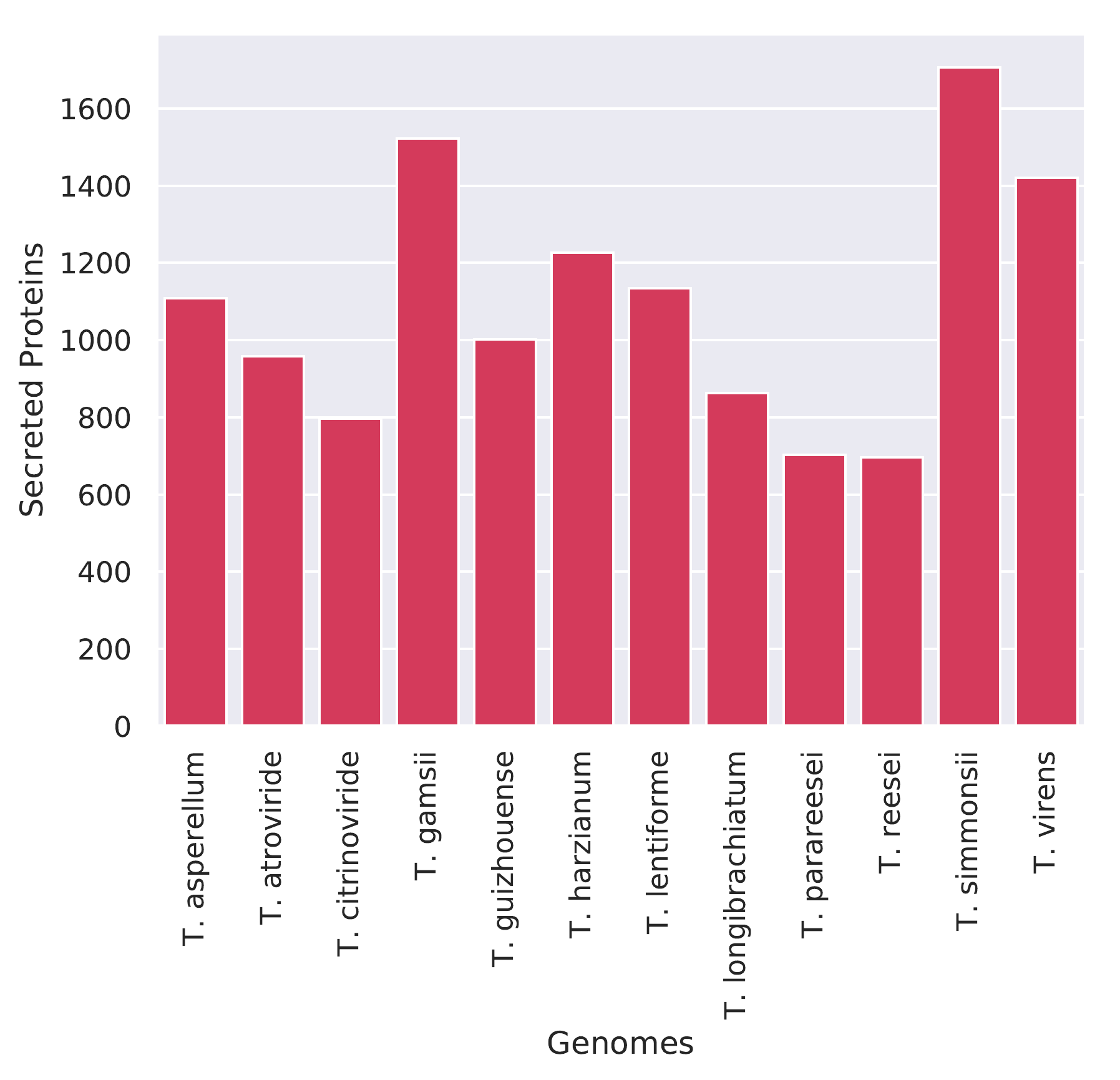


**Supplementary Figure 6. Signal peptides comparison among *Trichoderma* genomes.**

Funannotate pipeline predicted signal peptides from 12 *Trichoderma* genomes. *T. simmonsii* has the most secreted proteins (1,707) and *T. reesei* has the least (696).


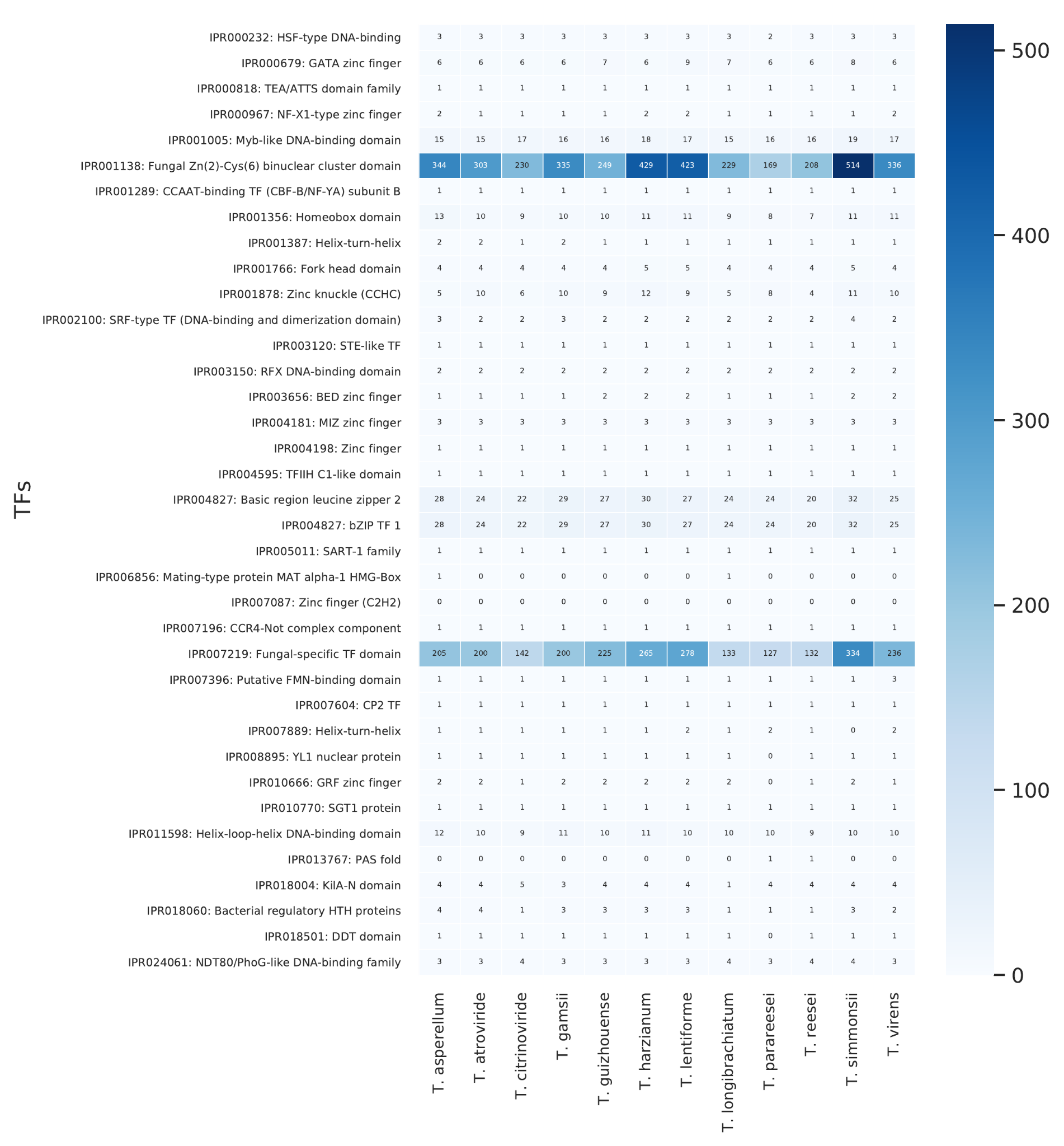


**Supplementary Figure 7. Abundances of transcription factors of *Trichoderma* genomes.**

Top 5 most abundant transcription factors in *T. simmonsii* are Fungal Zn(2)-Cys(6) binuclear cluster domain (514), Fungal-specific TF domain (334), Basic region leucine zipper 2 (32), bZIP TF 1 (32), and Myb-like DNA-binding domain (19).


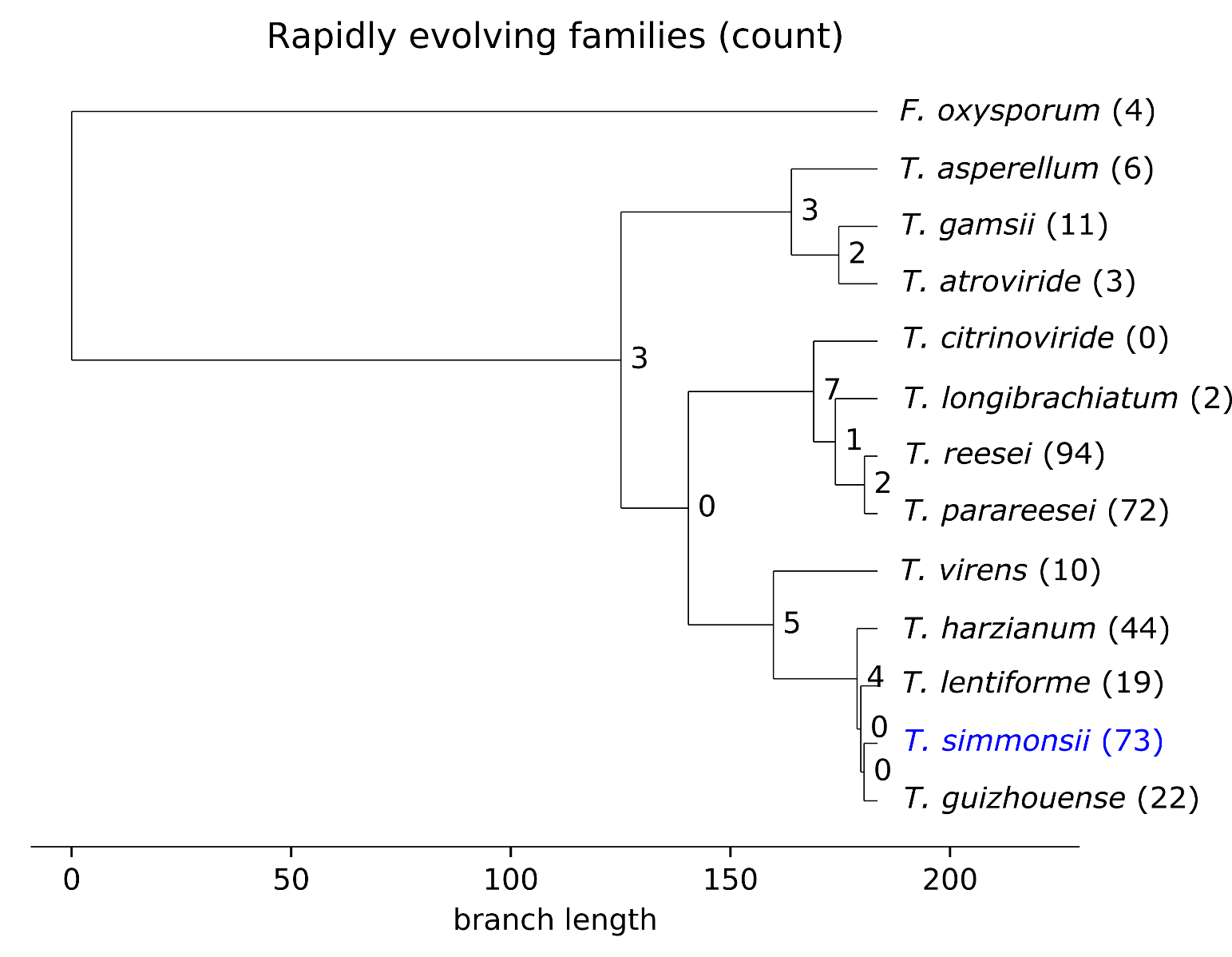


**Supplementary Figure 8. Rapidly evolving families of *Trichoderma* genomes.**

There are 73 rapidly evolving families in *T. simmonsii*, which is the second only to *T. reesei*, which has 94 such families.


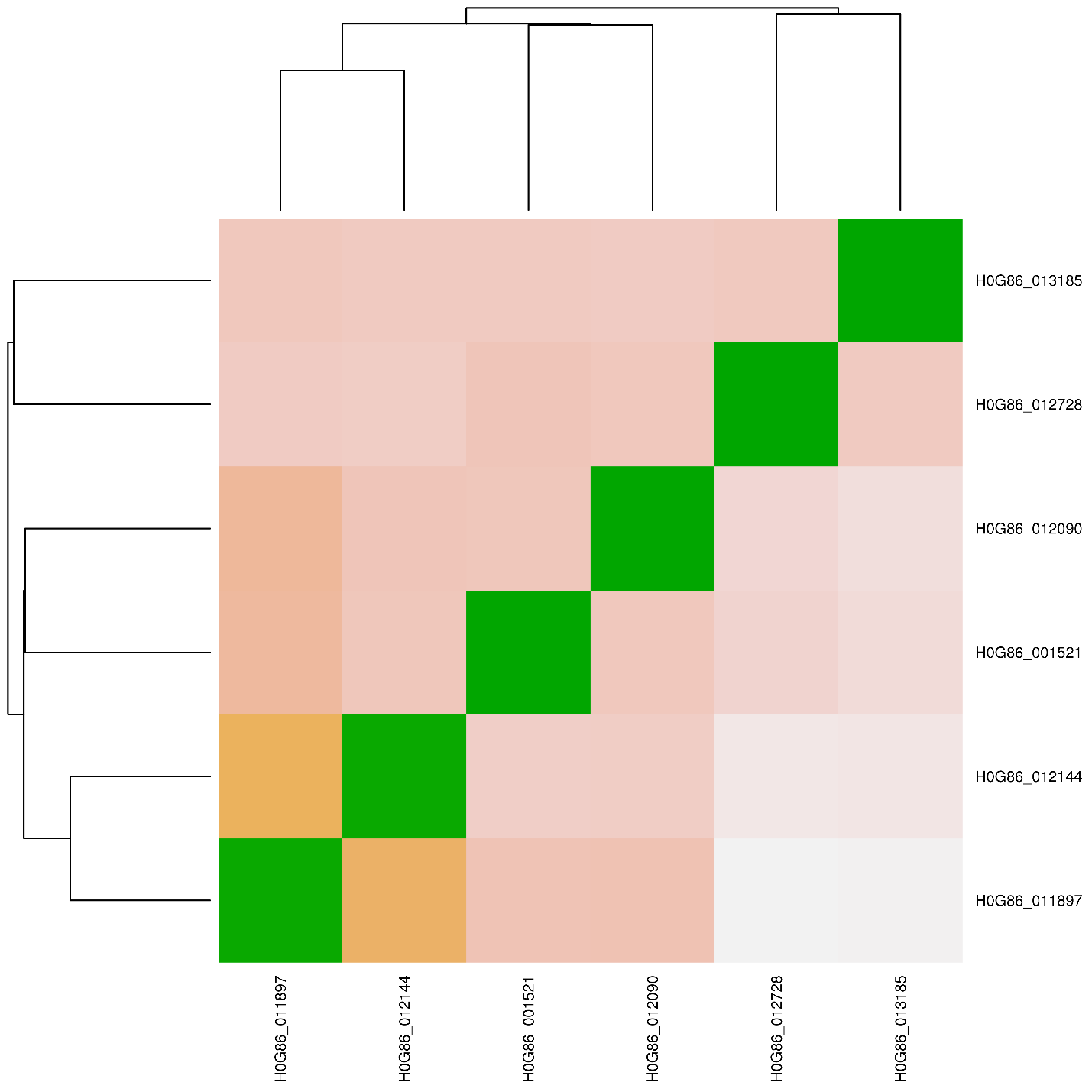


**Supplementary Figure 9. Pairwise comparisons of asparaginases**.

Six asparaginases were pair-wise aligned via BLASTP. E-values were used as distances in the heatmap, where e-value 100 was assigned in case no BLASTP matches reported. Four genes H0G86_001521, H0G86_011897, H0G86_012090, and H0G86_012144 forms a cluster, where the e-values are 1.15e-05 or lower. H0G86_012728 and H0G86_013185 do not have significant matches, where the lowest e-values are 0.22 and 3.0, respectively.


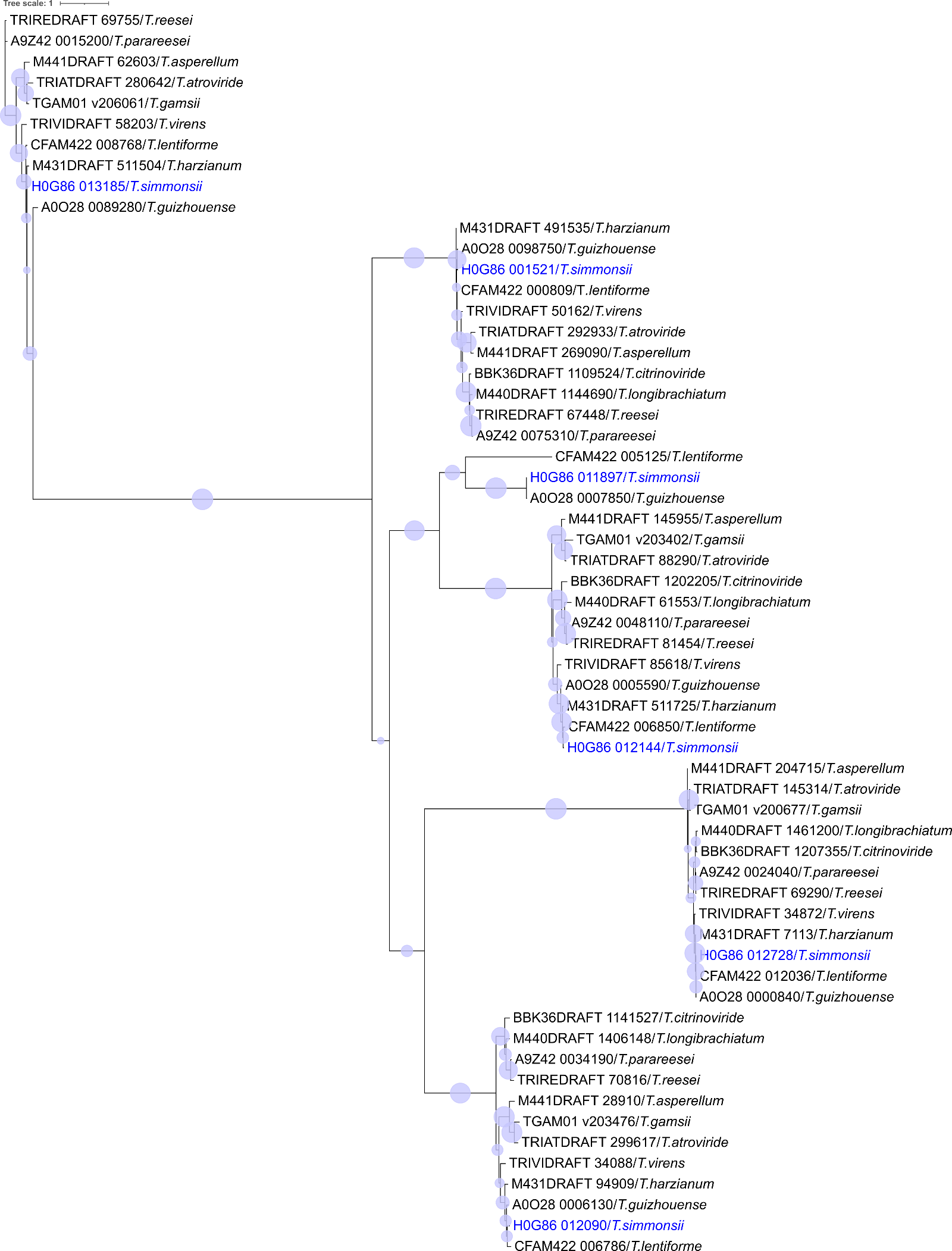


**Supplementary Figure 10. Asparaginase phylogeny of *Trichoderma* genomes.**

Phylogenetic tree of genes listed in Table 5 which produce asparaginase from 12 Trichoderma genomes were constructed. The size of circle on the nodes is proportional to the bootstrap supports. Genes in *T. simmonsii* are highlighted in blue.


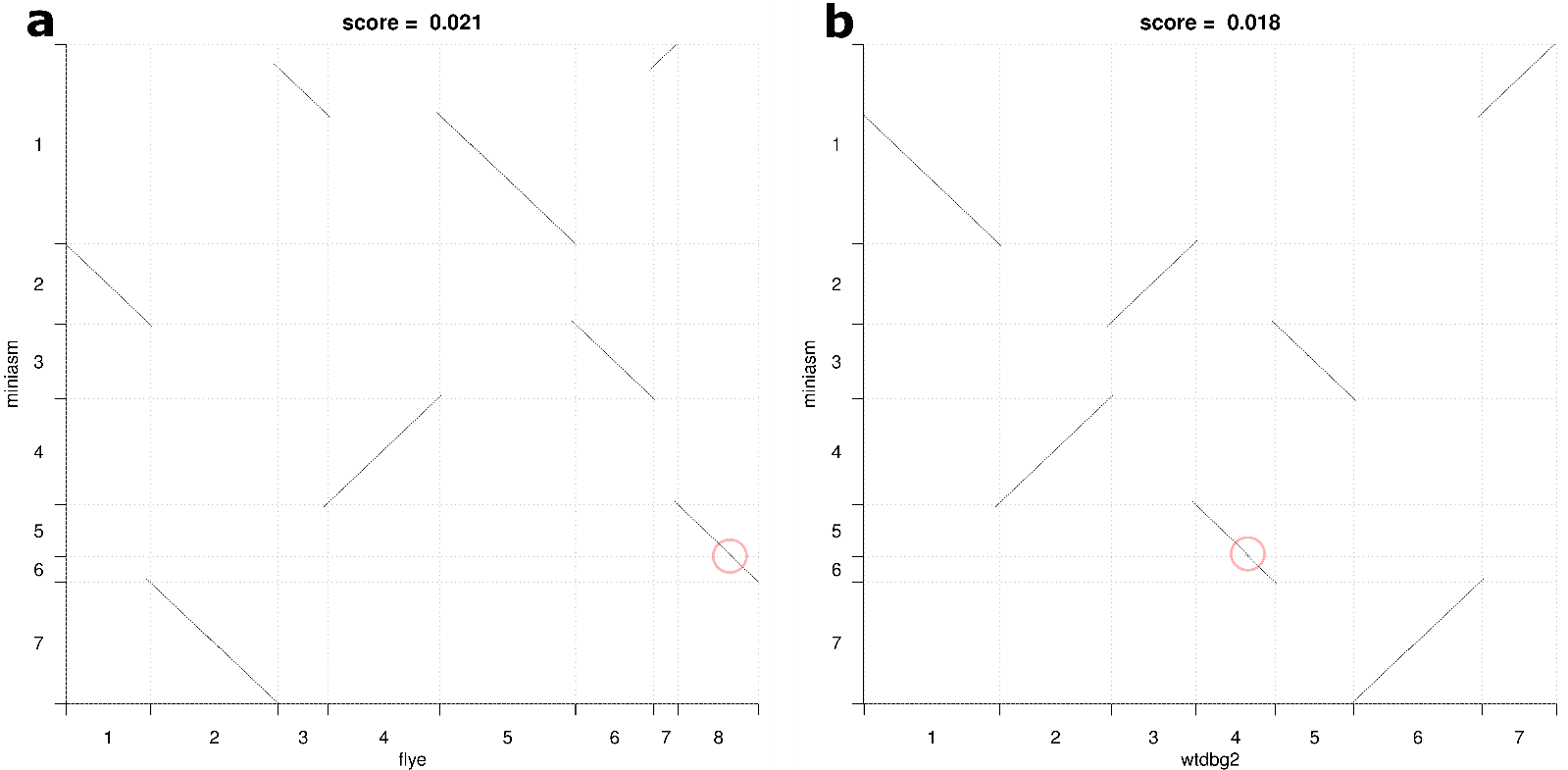


**Supplementary Figure 11. Pair-wise whole genome alignments.**

Each pairwise alignment and plot was generated using Chromeister. (a) whole genome alignment between Miniasm (y-axis) and Flye (x-axis). (b) whole genome alignment between Miniasm (y-axis) and Wtdbg2 (x-axis). The overlapping region of Miniasm contigs (5 and 6) were marked with red circles. Contig 5 and 6 of Miniasm assembly were assembled as contig 8 in Flye and contig 4 in Wtdbg2. For contig 1 of Miniasm, it was assembled to three contigs (3, 5, and 7) of Flye and two contigs (1, 7) of Wtdbg2.


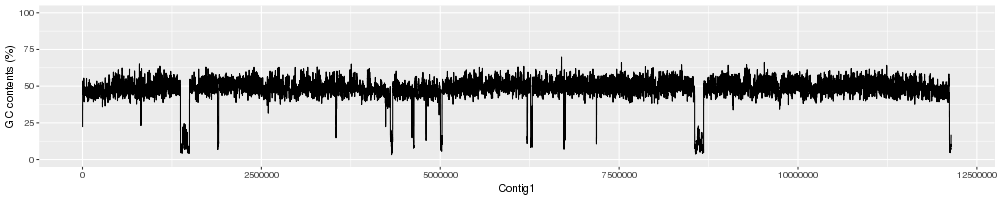


**Supplementary Figure 12. GC contents distribution on Contig1**.

GC contents of the contig 1 of Miniasm assembly were plotted in 1Kb windows. There are two very high AT-rich regions at ~1.36Mb and ~8.55Mb. Each block stretches to ~125Kb.
